# Facilitated sequence assembly using densely labeled optical DNA barcodes: A combinatorial auction approach

**DOI:** 10.1371/journal.pone.0193900

**Published:** 2018-03-09

**Authors:** Albertas Dvirnas, Christoffer Pichler, Callum L. Stewart, Saair Quaderi, Lena K. Nyberg, Vilhelm Müller, Santosh Kumar Bikkarolla, Erik Kristiansson, Linus Sandegren, Fredrik Westerlund, Tobias Ambjörnsson

**Affiliations:** 1 Department of Astronomy and Theoretical Physics, Lund University, Lund, Sweden; 2 Department of Biology and Biological Engineering, Chalmers University of Technology, Gothenburg, Sweden; 3 Department of Mathematical Sciences, Chalmers University of Technology/University of Gothenburg, Gothenburg, Sweden; 4 Department of Medical Biochemistry and Microbiology, Uppsala University, Uppsala, Sweden; University of Helsinki, FINLAND

## Abstract

The output from whole genome sequencing is a set of contigs, i.e. short non-overlapping DNA sequences (sizes 1-100 kilobasepairs). Piecing the contigs together is an especially difficult task for previously unsequenced DNA, and may not be feasible due to factors such as the lack of sufficient coverage or larger repetitive regions which generate gaps in the final sequence. Here we propose a new method for scaffolding such contigs. The proposed method uses densely labeled optical DNA barcodes from competitive binding experiments as scaffolds. On these scaffolds we position theoretical barcodes which are calculated from the contig sequences. This allows us to construct longer DNA sequences from the contig sequences. This proof-of-principle study extends previous studies which use sparsely labeled DNA barcodes for scaffolding purposes. Our method applies a probabilistic approach that allows us to discard “foreign” contigs from mixed samples with contigs from different types of DNA. We satisfy the contig non-overlap constraint by formulating the contig placement challenge as a combinatorial auction problem. Our exact algorithm for solving this problem reduces computational costs compared to previous methods in the combinatorial auction field. We demonstrate the usefulness of the proposed scaffolding method both for synthetic contigs and for contigs obtained using Illumina sequencing for a mixed sample with plasmid and chromosomal DNA.

## Introduction

Shotgun sequencing is the characterization of the genome of an organism by sequencing random DNA fragments and subsequently assembling the sequences *in silico*. The Human Genome Project was accomplished with first-generation sequencing, known as Sanger sequencing, which was the gold standard for two and a half decades [1]. Since the completion of the Human Genome Project, demands for cheaper and faster sequencing methods have driven the development of next-generation sequencing (NGS) [2] and third generation sequencing platforms [3]. Such platforms are massively parallel, allowing millions of fragments to be sequenced simultaneously. In such high-throughput sequencing, sufficient amounts of data to reconstruct the human genome can be obtained within a day.

The general problem with assembling long DNA sequences is that it is, in most cases, not possible to sequence a whole genome directly in one read. In Sanger sequencing, low-throughput long reads (800—1000 bps) are generated with high costs [1]. In contrast, NGS typically generate short reads with a length limited to 100-350 basepairs [4, 5]. Sequence assembly refers to the computational process of piecing all long reads (Sanger sequencing) or short reads (NGS) together to form longer contiguous sequences, contigs. A contig refers to a set of overlapping DNA segments that together represent a contiguous region of DNA, and is rather straightforward to assemble using bioinformatics tools [4, 5]. To obtain a complete genome sequence, contigs need to be merged into super-contigs (scaffolds), but since this step typically requires scaffolding information, it is not always feasible. In emerging sequencing platforms, such as PacBio sequencing [3], read lengths are longer, but they are also associated with a larger error rate and require DNA of higher quality [6].

In human genome analysis, the sequence assembly is aided by the reference provided through the Human Genome Project, which has paved the way for use of sequencing in forensics and diagnostics to mention a few examples. For organisms without a previously characterized genome, *de novo* assembly is required [7]. This process is often a difficult undertaking and provides no guarantee for a fully reconstructed genome. Indeed, for organisms with more complex genomes such as those containing high abundance of repetitive regions and/or high ploidity number, this process typically results in a high number of short contigs [4]. This is an inherent problem of NGS *de novo* sequence assembly due to the short read-length that cannot span repeats. To date, only a limited number of eukaryotic genome sequencing project has resulted in fully assembled genomes and even though prokaryotic genomes are much smaller, they frequently cannot be completely assembled using only short-read sequencing methods [8]. This calls for complementary methods that can provide scaffolding information [8].

In parallel to DNA sequencing efforts, optical DNA mapping has emerged as a method for characterization of long single DNA molecules [9]. Optical mapping of DNA was pioneered more than 20 years ago [10] and is based on coarse-grained visualization of the sequence of intact, ultra-long DNA molecules. While base-by-base NGS sequencing techniques suffer from short read lengths, there is no fundamental upper limit for the length of the DNA studied by optical mapping. For the present purpose, it is convenient to divide such mappings into two categories: (i) sparsely labeled optical maps, and (ii) densely labeled optical maps. Category (i) denotes cases where each label can be identified in the map and includes DNA fragments cut by restriction enzymes [10] (a label is here a cut position along the DNA), and sparse enzymatic nick-labeling [11, 12]. Contig scaffolding using type (i) optical maps was introduced in 1999, [13] employing restriction enzyme based methods. Later studies used sparsely distributed nick-labels [14]. These scaffolding methods utilizes either probabilistic frameworks [15–18] or more heuristic alternatives [19, 20]. The bioinformatics challenge associated with type (i) maps still attract interest [21–24]. The use of type (i) optical maps for contig scaffolding has resulted in two commercially available platforms: the OpGen Argus [25] and the BioNano Genomics Irys Systems [26], the latter of which was recently upgraded (the Saphyr platform). For type (ii) optical maps, the sequence-dependent DNA “fingerprint” is instead a continuous (amplitude modulated) signal along the DNA. Type (ii) approaches for DNA barcoding include melt mapping [27, 28], competitive binding, [29, 30] and dense enzymatic nick-labeling schemes [31].

In this proof-of-principle study we combine, for the first time, DNA sequencing and densely labeled experimental optical maps for addressing the challenge of bringing positional order to a set of contigs—contig scaffolding. On the experimental side, we use nanochannels to stretch single DNA molecules and obtain sequence-specific barcodes by using a competitive-binding scheme [29]. In this experimental assay a sequence-specific barcode is obtained by staining the DNA molecules with a mixture of YOYO and netropsin. Netropsin is a natural, non-fluorescent antibiotic with very high AT-specificity and outcompetes YOYO at AT-rich regions. This endows the DNA molecules with a barcode-like fluorescent profile based on the local AT/GC contents. In contrast to type (i) DNA barcodes, where each label is directly associated with a specific short DNA-sequence, type (ii) barcodes are less directly linked to DNA sequence. However, in [30] the necessary link between experimental barcodes and DNA sequence was provided by the transfer-matrix framework which here allows us to relate contig sequences to DNA barcodes.

The contigs used herein were obtained either by Illumina sequencing, one of the most used platforms for NGS, or by randomly cutting of previously sequenced DNA *in silico*. As proof-of principle, experimental DNA barcodes were obtained from four intact bacterial plasmids: pUUH239.2 (220 kilobasepairs (kbps)), pEC005A (70 kbps), pEC005B (138 kbps) and p4_2_1.1 (152 kbps). Since the chromosomal DNA is fragmented into shorter linear pieces with current sample preparation methods, it could not provide intact experimental DNA barcodes (the contig scaffolding method introduced herein requires intact experimental barcodes).

Plasmids can be replicated independently of the chromosomal DNA, can be transferred between bacterial cells, and are key players in the spread of antibiotic resistance among bacteria. Furthermore, due to the high density of transposable genetic elements and sequence repeats, plasmids are known to frequently undergo large scale rearrangements (translocations, inversions, copy number variations, insertions), making sequencing with short-read NGS methods particularly challenging [32]. For these reasons, the sequencing of plasmids serves as a good model to use for the evaluation of our method.

## Methods

Here, we introduce our method for contig scaffolding using densely labeled optical DNA maps. The problem of positioning plasmid contigs on a scaffold without overlap is formulated as a combinatorial auction problem. The input to the auction problem is a set of p-values for each contig. The use of a probabilistic method allows us to discard “foreign” contigs and thus deal with mixed contig samples from different types of DNA.

**Fig 1:**
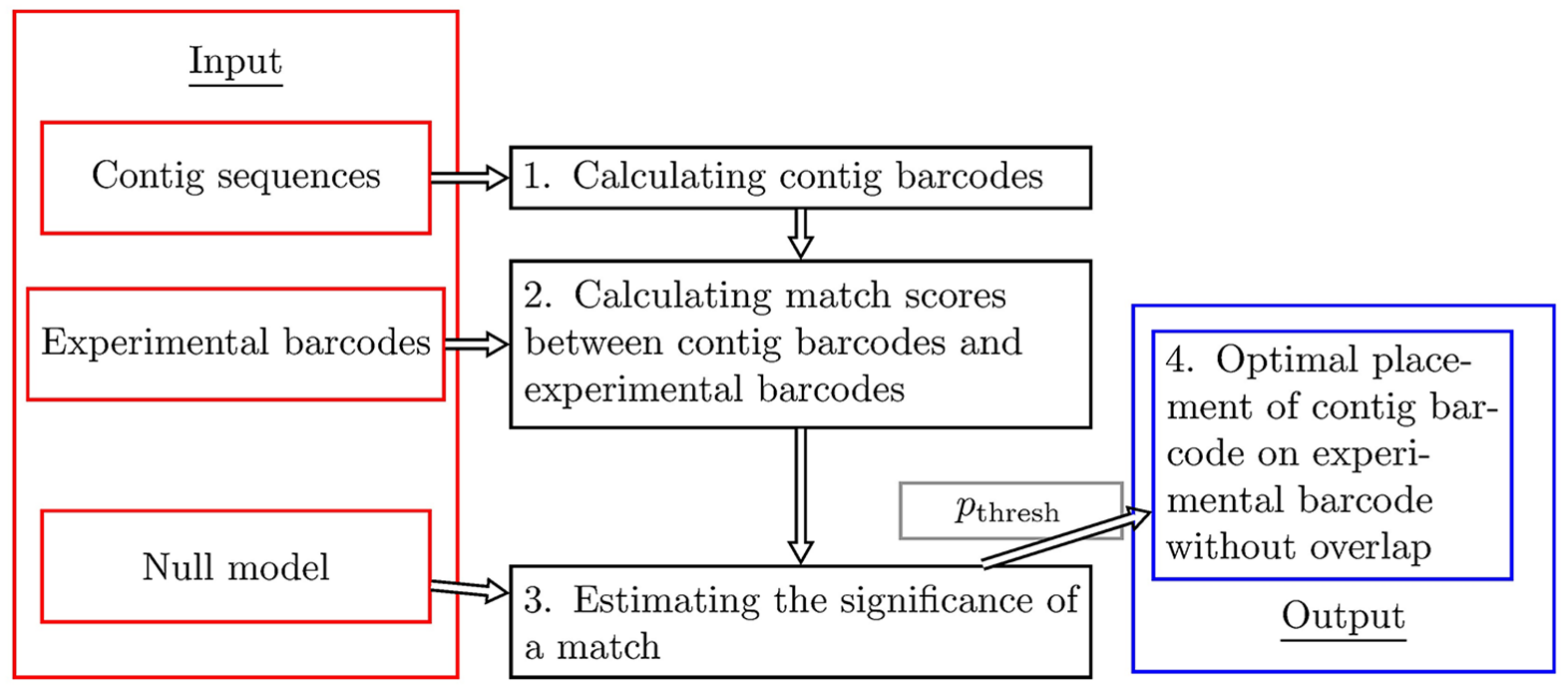
Schematic illustration of the four steps in our contig scaffolding method using optical DNA barcodes. On the left, we have the input to the method: a set of contig sequences, an experimental consensus DNA barcode (average over several single DNA barcodes), and a null model. Contig sequences are converted into theoretical contig barcodes, and compared to the experimental barcode by computing observed match scores for all positions (including flip, i.e., considering both orientations of the DNA barcode) along the experimental barcode. The null model is used to generate many random barcodes, and random-valued match scores between these random barcodes and the experimental barcode are then calculated. An extreme value distribution is subsequently fit to the histogram of random-valued match scores. Using this distribution fit, the observed match scores are converted into p-values, thus providing a significance level to each match. The p-values are in turn used to place the theoretical contig barcodes, using a method (combinatorial auction algorithm) which obeys the non-overlap constraint, on the experimental barcode. Our method also uses a p-value threshold, *p*_thresh_, to discard the barcodes that do not fit well on the experimental barcode.

As an input for our contig scaffolding method, we use *N* contig sequences and an experimental barcode. The experimental barcode is *x*_max_ pixels long. We here use experimental barcodes which are obtained by taking the average of repeated fluorescence measurements of the same type of DNA molecule (consensus barcodes, see Sec. S.M.2.2 in S1 Methods) [33]. We will henceforth refer to such averages simply as *experimental barcodes*. Our methods operate by converting the contig sequences into theoretical barcodes and, subsequently, placing these along the experimental barcode without overlap. We here use the name *contig barcodes* for such contig-based theoretical barcodes. The schematic illustration of our method is given in Fig 1.

The method can be summarized into four main steps:

1. Calculating contig barcodes. All contig sequences are converted into contig barcodes using competitive binding theory, see Sec. S.M.2.3 in S1 Methods and [30]. Briefly, the input to the calculation is a contig DNA sequence together with the total concentrations of the two types of ligands: the fluorescent molecule (YOYO-1) and the AT-specific ligand (netropsin), as well as the total concentration of DNA used in experiments. Additionally, a set of ligand binding constants are required for the computation of theoretical barcodes. We here use a refined set of such constants compared to [30] (see Sec. S.M.2.3 in S1 Methods and S1–S3 Figs, for details). The new set of netropsin binding constants are provided as a Supplementary text-file, see S1 File. Based on these constants, the probability that YOYO-1 is bound to DNA is calculated for each base-pair. This probability vector is then convolved with a Gaussian kernel with an experimentally determined standard deviation *σ* [30] to mimic the Point Spread Function (PSF) of the experimental assay, see Sec. S.M.2.4-2.5 in S1 Methods for more details. The result is finally interpolated down to pixel resolution, thus producing a contig barcode.
2. Calculating match scores between contig barcodes and experimental barcodes. Contig barcodes, here labeled by *n* (1 ≤ *n* ≤ *N*), are compared against the experimental barcode by computing observed match scores for all possible positions (including flips) of the contig barcodes, see Sec. S.M.2.6 in S1 Methods. This is done by “sliding” the contig barcode along the experimental barcode. For each starting position, and both directions (forward, 1 ≤ *x* ≤ *x*_max_, and backward *x*_max_ + 1 ≤ *x* ≤ 2*x*_max_), a Pearson correlation coefficient *C*_*n*,*x*_ is calculated (the orientation of contig sequences is not known). This gives us 2*x*_max_ match scores per contig. The maximum observed match score for the *n*th contig is denoted by a “hat”, i.e. ${\hat{C}}_{n}=\max_{x}\left[C_{n,x}\right]$.
3. Estimating the significance of a match. For contig barcodes longer than a length threshold *l*_thresh_ (see below), match scores are turned into p-values using a probabilistic method (similar to [34]). To that end, *R* (here *R* = 1000) random contig barcodes are generated based on their estimated correlation coefficient, see Sec. S.M.2.7 in S1 Methods for details. We then compute match scores between the random contig barcodes and the experimental barcode and store the maximum scores, ${\hat{C}}_{r}^{\left(\text{random}\right)}$ (*r* = 1, … *R*). A parametric probability density, $\phi (\hat{C})$, is fitted to the histogram for the ${\hat{C}}_{r}^{\left(\text{random}\right)}$ (see Secs. S.M.2.8 and S.M.2.9 in S1 Methods). Finally, *C*_*n*,*x*_ are converted to p-values using the distribution for $\hat{C}$: $p_{n,x}=1-\int_{-1}^{C_{n,x}}\phi \left(\hat{C}\right)d\hat{C}$, see S4 Fig for an example. A match of a contig barcode is considered to be significant if its observed p-value, *p*_*n*,*x*_, is smaller than *p*_thresh_ = 0.01 [33]. Thus, we accept no more than 1% misallocations.
4. Optimal placement of contig barcodes on an experimental barcode without overlap. Contig barcodes are finally placed, obeying the non-overlap constraint, on experimental DNA barcodes. For each contig 1 ≤ *n* ≤ *N*, and for all possible locations 1 ≤ *x* ≤ 2*x*_max_, we define a placement score *b*_*n*,*x*_
  $$\begin{array}{c} b_{n,x}=\left\{ \begin{array}{c} -2\log\left(p_{n,x}\right),\;p_{n,x}<p_{\text{thresh}}\\ 0,\;\text{otherwise.} \end{array}\right. \end{array}$$ (1)
  Note that *b*_*n*,*x*_ ≥ 0 by construction. Since we are interested in placing several contigs at the same time without overlap, we calculate an overall placement score for a given set of contigs and their placements, by summing the individual placement scores. In mathematical terms, let *y*_*n*,*x*_ = 1 if contig *n* is included in the final contig placement at the location *x*, and *y*_*n*,*x*_ = 0 if it is not. Then the contig scaffolding problem is here formulated as the following global optimization problem:
  $$\begin{array}{c} F_{\text{overall}}\left(y\right)=\max\sum_{n=1}^{N}\sum_{x=1}^{x_{\text{max}}}b_{n,x}y_{n,x} \end{array}$$ (2)
  The problem becomes non-trivial due to the constraints that a contig can be placed at most once, and that contigs cannot overlap. In practice, the global optimization problem above is solved using a combinatorial auction algorithm [35], see Sec. S.M.3 in S1 Methods, which guarantees that each pixel is covered by at most one contig. Note that due to the non-overlapping constraint, a contig, if placed, is not necessarily placed where it fits best if another contig has a higher placement score when placed at that pixel.

In step 3, for consistency in our approach, a length threshold *l*_thresh_ is introduced. Since the spatial resolution in optical mapping experiments is set by *σ* (width of the PSF), a barcode must be several *σ*s long to contain meaningful spatial information. We here choose *l*_thresh_ = 12*σ* ≈ 12 kbps. This choice also guarantees that the parametric probability density $\phi (\hat{C})$ fits well for all contig lengths considered, see S5 Fig.

## Results

Here, we present the results of our contig scaffolding method applied to synthetic and Illumina contigs.

### Illumina contig alignment on reference sequences

We start by aligning the Illumina contig sequences to the known full DNA sequences. These alignments later serve as as a means of validating our method. There are a variety of bioinformatics tools that can place nucleotide sequences along a reference sequence. In the cases at hand, the plasmids and chromosomal DNA have been sequenced previously (see Sec. S.M.1.2 in S1 Methods), so finding the best position for each contig is reasonably easy with any local sequence alignment tool. MUMmer [36], a tool designed to find maximal exact matches between sequences, is used here.

We find (see Sec. S.M.1.3 in S1 Methods) that out of the 220 contig sequences that we have, 203 belong to the chromosomal DNA (we name these *CX*, *X* = 1, … 203), 16 to the plasmid (*PX*, *X* = 1, … 16), and one contig is foreign (*U*1).

### Validation of input parameters and p-value

To make theory and experimental barcodes as similar as possible, we refined previously used ligand binding constants using experimental barcodes for the pEC005A and pEC005B plasmids as input. We find that the new binding constants improve the $\hat{C}$ values by 0.02-0.07 compared to the old values [30] (see S1 Table).

In order to validate our method for calculating p-values, we cut out synthetic (artificially generated) contigs of different lengths from the pEC005B plasmid sequence and find the fraction of correctly placed contigs by comparing them separately to experimental barcodes for pEC005B (thus, no combinatorial auction algorithm is used). We find that at the chosen value for *p*_thresh_(= 0.01), we get ≈ 1% misallocations (for contigs longer than *l*_thresh_), as it should (see S6 Fig).

### Scaffolding of pure pUUH samples with synthetic contigs

**Fig 2:**
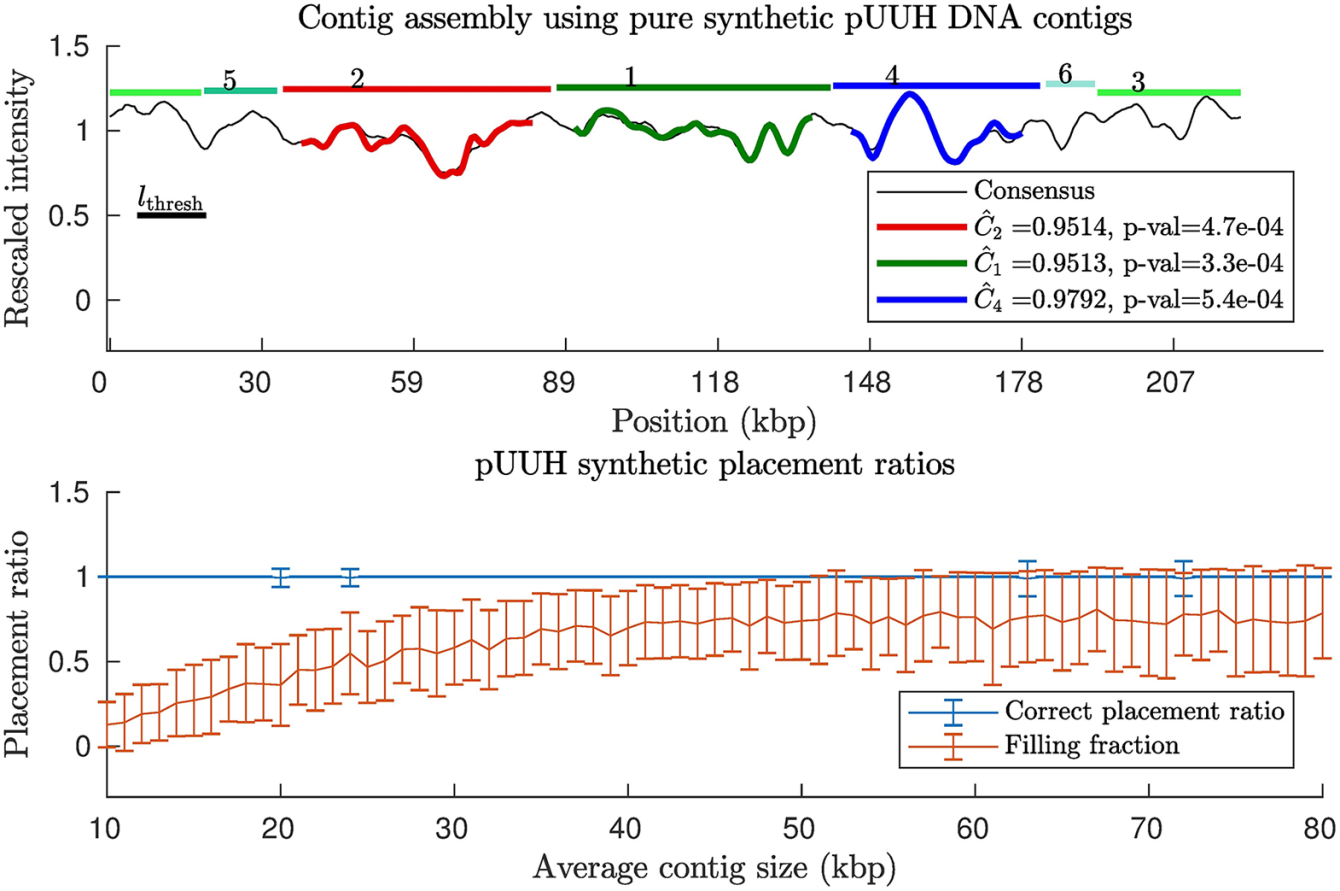
Contig scaffolding using synthetic contigs from a pure pUUH contig sample (no chromosomal DNA). Synthetic contigs were generated by randomly cutting the known DNA sequence for the pUUH plasmid. The distances between cuts were taken from a truncated exponential distribution with average sizes varying from 10 kbps to 80 kbps. We then applied our contig scaffolding method (see Methods). (Top) Example of contig barcodes assembled on the consensus pUUH barcode, here with average contig size = 24.5 kbps. (Bottom) Two placement ratios: the filling fraction = number of occupied pixels/total number of pixels in experimental barcode, and correct placement ratio = number of correctly placed contigs/total number of contigs. This was repeated for 100 random realizations of the cutting process, and mean values and associated standard deviations for these ratios were calculated. A similar plot for the p4_2_1.1 plasmid is found in S8 Fig.

To gauge the sensitivity of our p-value and combinatorial auction based contig scaffolding method to changes in contig size and to the non-overlapping constraint, we created synthetic contigs from the full pUUH sequence but with no chromosomal DNA (applying our method to a pure sample with real pUUH contigs *PX* (*X* = 1, …, 16). The synthetic pUUH contigs were obtained by cutting the pUUH sequence into randomly sized contigs following a truncated exponential distribution (see S7 Fig) with different mean lengths. We always truncate the distribution at the length of the sequence since contigs are assumed not to be longer than the sequence itself. Our method was then applied, with results shown in Fig 2. We see that for all contig sizes we are able to place the contigs with a success rate close to the expected 1 − *p*_thresh_ = 99%. The filling fraction, i.e., the number of pixels which were occupied after contig placement divided by total number of pixels in the experimental barcode, were found to be in the range 0 to 26% within one standard deviation from the mean for the shortest contigs considered, and for the longest contigs filling fractions range between 52 and 100%. For synthetic contigs from the p4_2_1.1 plasmid we find very similar results, see S8 Fig.

### Scaffolding of a mixed pUUH/chromosomal sample with synthetic contigs

We now investigate contig size dependencies of our scaffolding method for a mixed sample with pUUH and chromosomal contigs. This problem is more challenging than dealing with a pure plasmid sample. In particular, the chromosomal DNA is much longer than the pUUH plasmid DNA, and as a result there are roughly one order of magnitude more chromosomal contigs than there are plasmid contigs.

**Fig 3:**
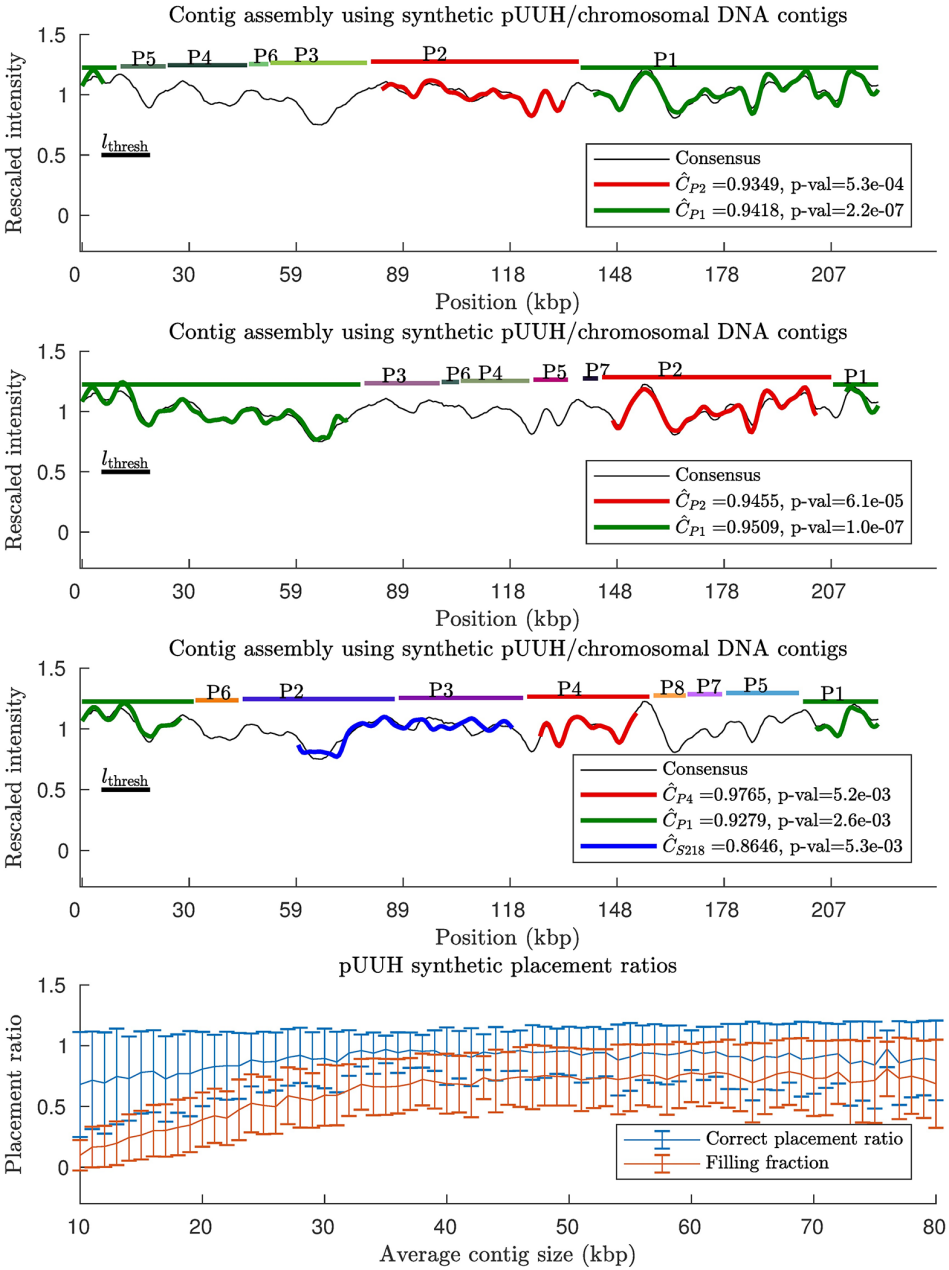
Contig scaffolding using synthetic contigs from a mixed sample of pUUH/chromosomal DNA. Synthetic contigs were generated by randomly cutting the known DNA sequences for pUUH and the chromosomal DNA from *Klebsiella pneumoniae*. The distances between cuts were taken from a truncated exponential distribution. We then applied our contig scaffolding method (see Methods). (Top) Three typical examples of contig barcodes assembled on the consensus pUUH barcode, here with average contig size = 24.5 kbps. In the first two examples all placed contigs end up at correct positions, whereas in the third example there is one misplaced contig barcode. (Bottom) The two ratios: the filling fraction = number of occupied pixels/total number of pixels, and the number of correctly placed contigs/total number of contigs were calculated. This was repeated for 100 random realizations of the cutting process, and mean values and associated standard deviations were calculated. We find that our method is effective at separating chromosomal and pUUH DNA and, also, it rarely places a contig at the wrong position. The filling fraction increases with increasing contig size. A similar plot for the p4_2_1.1 plasmid is found in S11 Fig.

As in the previous subsection, we generate synthetic contigs by randomly cutting the DNA sequences, where the distance between cuts are taken from a truncated exponential distribution with varying average size. Our method was then applied, with results shown in Fig 3. As for the pure pUUH sample, we are able to place most of the contigs at correct places and our method is effective at discarding chromosomal DNA (for all contig sizes above the length threshold). Note, however, that for this mixed sample, the ratio of correct placements to total number of placements is below 1 − *p*_thres_ = 99% on average. The reason for this, rather minor, effect is that typically a few chromosomal DNA contigs fit sufficiently well in the gaps which remain after the pUUH contigs have been placed. To investigate this false positive effect further, S9 Fig shows the placement of chromosomal contigs on the pUUH sequence (no pUUH contigs), where we find that the fraction of placed contigs is close to the expected *p*_thres_ = 1%. For synthetic contigs from the p4_2_1.1 plasmid we find very similar results, see S10 Fig. Since the average number of chromosomal contigs is large, 100 − 140 contigs pass the length threshold *l*_thresh_ for the contig sizes considered in Fig 3, this corresponds to roughly to 0 − 2 falsely placed contigs at the significance level used herein (*p*_thres_ = 0.01). Filling fractions are similar to the results obtained for a pure pUUH sample (see previous subsection).

### Scaffolding of real contigs for a mixed pUUH/chromosomal DNA sample

**Fig 4:**
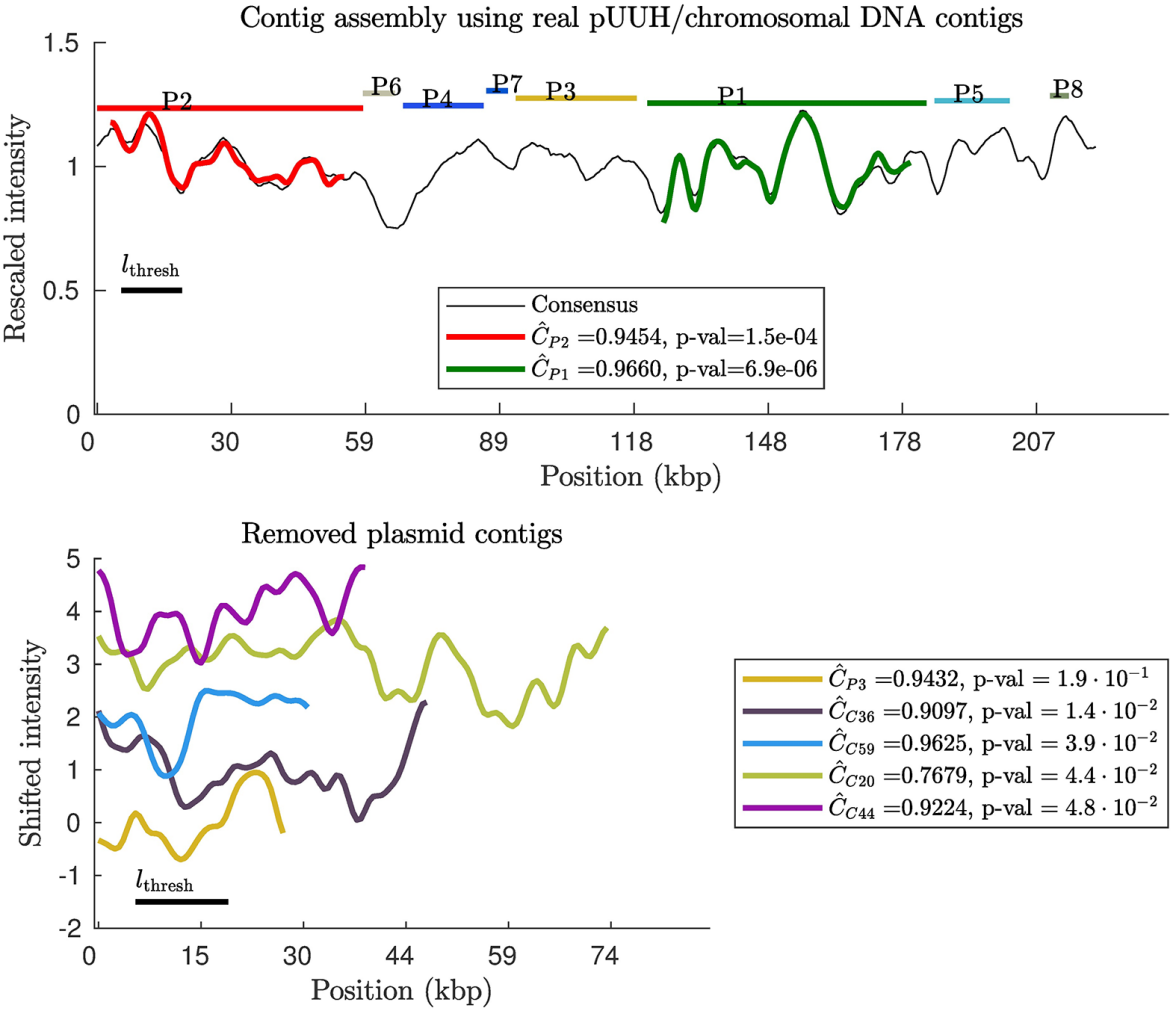
Contig scaffolding using Illumina contigs from a mixed sample of pUUH/chromosomal DNA. (Top) Optimal placement of the contig theory barcodes on the experimental pUUH barcode using our contig scaffolding method (see Methods). 220 contigs were obtained through Illumina sequencing of a mixed sample containing the pUUH plasmid and chromosomal DNA from the bacterium *Klebsiella pneumoniae*. Based on a sequence alignment 16 of the contigs are deemed to belong to the pUUH plasmid. Horizontal lines at the top corresponds to “true” contig positions based on a sequence comparison of the full pUUH sequence and the contig sequences. We find that 2 contig barcodes pass the length and p-value thresholds. The two contigs which were placed ended up at correct positions. (Bottom) The examples of removed contigs illustrates intensity profiles of a few typical non-matching barcodes: the four chromosomal contig barcodes with the smallest p-values and the third longest plasmid barcode (orange).

We finally turn to real contigs from a mixed sample. With the 220 Illumina contigs and the experimental pUUH barcode as input, we applied our contig scaffolding approach. We know that 16 contigs are plasmid contigs and 203 are chromosomal contigs, as described previously. When placed on the experimental barcode, only 2 passed the p-value threshold (= 0.01). The placement of these contigs on the experimental barcode is shown in Fig 4. The two contigs (contigs *P*1, *P*2) end up at their correct positions. Notice also that the third largest plasmid contig (*P*3), see Fig 4(bottom), has a large correlation coefficient, but does not yield a sufficiently small p-value to be placed (for small contig barcodes, large values for the correlation can occur by chance). In conclusion, our method is successful at separating chromosomal and plasmid contigs with 1% error rate. Furthermore, our method was able to correctly place the plasmid contigs.

## Discussion

Below we make some more technical comments on some computational aspects of our new combinatorial auction algorithm and briefly discuss how one in the future may also scaffold chromosomal DNA using optical DNA maps.

In the spirit of Fisher’s method for combining p-values [37], our overall placement score is obtained by summing the individual placement scores, see Eqs (1) and (2). In order to calculate the placement score we use p-values, which in turn requires the distribution for maximum of the Pearson correlation coefficient. This distribution is known for the maximum correlation coefficient between two sets of independent Gaussian random numbers, see Sec. S.M.3.6 in S1 Methods. However, since pixels are correlated along the DNA barcode [38], we have to replace the parameters in this distribution by effective ones in a similar spirit as was done in [30]. We find that this works well in practice, except for when the contig barcodes becomes smaller than *l*_thresh_.

The barcodes which are remaining after step 3 in our contig scaffolding approach (see Methods) need to be placed on an experimental barcode in the optimal way (step 4). For each contig *n*, which passed the p-value threshold, we assign scores dependent on position and flip, −2 log(*p*_*n*,*x*_). Our overall score is then obtained by summing the individual placement scores. This choice of overall placement score is inspired by Fisher’s method for combining independent p-values [37]. Note, however, that in our case the p-values for different contig barcodes are not necessarilary independent, and one should therefore view our choice of overall score simply as a convenient score for our purposes: the quantity −2 log(*p*_*n*,*x*_) is, by construction, positive (since 0 ≤ *p*_*n*,*x*_ ≤ 1) as required in the combinatorial auction algorithm (see below).

In order to find the maximum overall placement score, we use a combinatorial auction algorithm. In the terminology of combinatorial auctions, a contig is a “bidder” who places bids for sets of “items” (pixels). In our case, a bidder only bids for consecutive items and bids can therefore be labeled by *b*_*n*,*x*_, where *n* labels bidders and *x* is the last item in the consecutive set of items which are bid for, see S12 Fig. Problems involving only bids for consecutive items are called interval bidding auctions [39, 40]. In Sec. S.M.3 in S1 Methods we provide a computationally improved version of the exact algorithm from [39] for solving the Combinatorial Auction (interval bidding) problem. Our method extends the dynamical programming method in [39] in two ways: (i) no extra computational time is spent where there are gaps in-between bids (i.e. regions where no bids are placed), and (ii) at a given stage in the dynamical programming method, we only include “relevant” subsets of bidders. The computational times is expected to scale as *AB*^2^2^*C*^, where for the method in [39] estimated parameters are *A* = *N*, *B* = *x*_max_, *C* = *N* with *x*_max_ signifying the number of items (number of pixels in the experimental barcode) and *N* signifying the number of bidders (number of contigs). In our case, instead, *A* ≤ *N*, *B* ≤ *x*_max_, C=log2(∑k=1D(Nk))≤xmax, where *D* < *N*. The reduction in the exponent *C* can, in practice, be rather large, see Sec. S.M.4.5 in S1 Methods.

It should be noted that the main computational cost of our method results from the non-overlapping constraint for DNA fragments (contigs). This constraint leads to a non-linear dependence of the computational time on the number of fragments. For cases when this constraint is not needed, the computational time instead scales linearly with the number of fragments.

We use experimental consensus barcodes, obtained by averaging several individual DNA barcodes, for scaffolding. The current method for creating consensus barcodes [33] requires the individual DNA barcodes to originate from intact (non-fragmented and circular) DNA molecules. As a result, here we only choose to scaffold plasmid contigs (the chromosomal DNA is fragmented into shorter linear pieces with current sample preparation methods). It remains a future theoretical challenge to develop methods for creating consensus barcodes from fragmented DNA. If such a methodological development is successful, one should be able to also scaffold contigs from the chromosome.

## Conclusions and outloook

We demonstrated here that it is possible to partially or fully piece together contigs using densely labeled competitive binding DNA barcodes as a scaffold. Our procedure is expected to be of general use for any densely-labeled optical maps, such as DNA melting maps [27, 28] or DNA with dense covalent labels [31]. Note, however, that the step where we generate random DNA barcodes may need to be adapted to the particular choice of experimental assay.

Our probabilistic approach uses a p-value threshold which was set to 1% here as in our previous study [34]. In the validation part of this study, it is demonstrated that this choice of threshold leads to the expected error rate of 1% for contig placement. In applications where a different error rate is preferable for contig placements, one can simply tune the p-value threshold accordingly. Since we are using a probabilistic approach, “foreign” contigs tend to be automatically discarded. We showed that this feature of our method allowed us to successfully process a mixed contig sample which contained both plasmid and chromosomal DNA.

Herein, we used contigs obtained from the Illumina sequencing platform. There are other platforms, such as PacBio and nanopore sequencing, which, typically, produce longer contigs. As the present method is indifferent to the origin of the contigs, it can also be directly applied to contigs from these other sequencing platforms.

A fundamental limitation of the contig scaffolding is the width of the optical point spread function (≈ 1 kbps) for the current experimental assay. This resolution limit sets a sharp lower bound the lengths of contigs one will be able to assemble using the present method. However, in the future optical mappings using super-resolution methods [41] could potentially enable the scaffolding of shorter DNA fragments with our method. Also, very short contigs can potentially be positioned using gene-specific labels on the optical map, such as labels obtained by using the CRISPR/Cas9 system [42, 43].

We expect the present methodology to serve as a complement, or sometimes a replacement, for similar scaffolding effort using sparsely-labeled DNA molecules, where commercial platforms are available (OpGen Argus and BioNanoGenomics Irys and Sapphire Systems). A benefit of the competitive binding scheme is that involves only simple pipetting and does not require extensive washing of the samples which makes sample preparation more straightforward compared to the BioNanoGenomics assay. Moreover, the competitive binding assay could serve as an add-on to the BioNanoGenomics platform—this platform utilizes YOYO to stain and subsequently identify the DNA molecules and by simultaneously adding netropsin to their samples one would obtain extra sequence information, without increased complexity of the experimental setup.

## Supporting information

S1 Methods Supplementary methods. Here we provide details and computational/mathematical arguments for the methods we use, together with examples.
(PDF)

S1 TableComparison between match scores (maximum Pearson correlation coefficients, $\hat{C}$) using the new competitive binding parameters and the old method from [30].(PDF)

S1 Fig Free concentration as a function of total concentrations of YOYO-1 and netropsin. Free concentrations (concentration of ligands which are not bound to DNA) were computed for YOYO-1 binding constant *K*_1_ = 26 *μM*^−1^, by a minimisation procedure described in Supplementary Methods. Note that (Top) the total YOYO-1 concentration is always larger than the corresponding free concentration, and the difference between the two increases as we increase the YOYO-1 concentration. Here concentrations are chosen in a range typical for optical DNA mapping experiments. (Bottom) As more and more YOYO-1 is bound to DNA, the free concentration of netropsin increases.
(EPS)

S2 Fig YOYO-1 binding constant optimization. We find the optimal YOYO-1 binding constant by minimizing the sum square differences between the pEC005 experimental barcodes and the corresponding theory barcodes. Note that there is a range of possible binding constants. Our selected value for YOYO-1 binding constant, *K*_1_ = 26 *μM*^−1^, lies close to the minima of the two sum squared curves, but any value between ≈10 *μM*^−1^ and 40 *μM*^−1^ would give very similar results.
(EPS)

S3 Fig Improved netropsin binding constant obtained using literature fluorescence data. (Left) Percentage fluorescence values were extracted from the Supplementary figures from [Boger DL, et al. A simple, high-resolution method for establishing DNA binding affinity and sequence selectivity. Journal of the American Chemical Society 2001;123(25):5878-5891]. Lower fluorescence corresponds to higher netropsin binding. Based on the extracted fluorescence, netropsin binding constant were subsequently extracted using a procedure described in Supplementary Methods and provided as a Supplementary File S1 File. The resulting binding constants are then plotted against AT content (Right). Blue crosses contain G or C residues in at least one of the two central positions of the 4-mer. The red circles correspond to 4-mers that have A or T residues in both central positions. Those 4-mers with 3 AT residues which also contain no central C or G residue have a higher binding constant than the ones that do have a central C or G. This is not seen for 4-mers with 2 AT residues.
(EPS)

S4 FigIllustration of our p-value thresholding procedure for contig selection.We created *N* = 250 synthetic contigs, each of length *M* kbps, from the pEC005B DNA sequence. We here used *M* = 20, 30, 40, 70 kbps. These associated contig barcodes were then compared to the pEC005B experimental barcode, and corresponding p-values were computed. Note that only the contigs whose maximum match score ${\hat{C}}_{n}$ is above the gray solid line (p-value threshold = 0.01) are placed (*n* labels different contigs). The green dots depict the contigs who have ${\hat{C}}_{n}$ at the correct place, and the red dots depict the contigs that have ${\hat{C}}_{n}$ at the wrong place (based on sequence information).(EPS)

S5 FigExamples of the distribution fit for maximum match scores.Here, 1000 random barcodes of lengths 10 (top, left), 20 (top, right), 30 (bottom, left) and 40 (bottom, right) kbps were generated and compared to the pUUH experimental barcode using the match score, $\hat{C}$. The match score histograms were then fitted using maximum likelihood to (i) our new proposed functional form (in blue) and to (ii) a Gumbel PDF (in red). With increasing contig barcode length, the mean of the PDF is shifted to smaller $\hat{C}$-values (note that the upper two and the lower two plots have different ranges of $\hat{C}$ values). Note that our new functional form fits better than the previously used Gumbel PDF in all cases.(EPS)

S6 Fig Placement ratio statistics for synthetic contig placement on pEC005B and on p4_2_1.1. The pEC005B and p4_2_1.1 sequences were cut into *N* equally sized contigs of lengths ranging from 12 kbps to 65 kbps. Then, for each contig separately (skipping the combinatorial auction step), we applied our contig scaffolding method (see the Methods section in the main text), and contigs were labeled as unplaced, placed at correct position, or placed at an incorrect position. Based on this data, the two placement ratios: the ratio of number of placed contigs/total number of contigs and the number of correctly placed contigs/total number of contigs were calculated. Note that for lengths ≈12 kbps for pEC005B and ≈14 kbps for p4_2_1.1, no contigs are placed due to our p-value threshold.
(EPS)

S7 Fig Histograms of contig lengths from a sample containing pUUH and chromosomal DNA from Illumina sequencing. (Top) Histogram of the length of real Illumina contigs from a sample containing pUUH and chromosomal DNA. Fits were done using moment matching, i.e., obtained by equating the mean of the PDF to the sample mean of the data. Note that the histogram follows the exponential distribution. The mean contig size in this data set was 24.5 kbps. (Bottom) Histogram of synthetic contig lengths from a mixed sample containing pUUH/chromosomal DNA.
(EPS)

S8 Fig Contig scaffolding using synthetic contigs from a pure synthetic sample of p4_2_1.1 plasmid DNA. (top) Optimal placement of the contig theory barcodes on the experimental p4_2_1.1 barcode using our contig scaffolding method (see Methods). The contigs were generated using a procedure identical to the associated pUUH sample, see Fig 2 in the main text. Two placement ratios: the filling fraction = number of occupied pixels/total number of pixels in experimental barcode, and correct placement ratio = number of correctly placed contigs/total number of contigs. This was repeated for 100 random realizations of the cutting process, and mean values and associated standard deviation for these ratios were calculated.
(EPS)

S9 Fig Placement statistics for synthetic contigs from the chromosomal DNA on the pUUH experimental barcode. We created synthetic contigs from the *Klebsiella pneumoniae* chromosomal sequence, by cutting at random positions. Distances between cuts were taken from a truncated exponential distribution. Attempts were made to place these these synthetic chromosomal contigs on the pUUH experimental barcode using our contig scaffolding method. The placement ratio, i.e., the number of placed contigs/total number of contigs was calculated, for different mean contig sizes in the range from 10 kbps to 45 kbps. This procedure was repeated 100 times, providing us with mean and standard deviations for the placement ratios. If chromosomal DNA and pUUH experimental barcodes had no sequence similarity, we would expect the placement ratio to be around *p*_thresh_ = 0.01, which is generally consistent with our findings.
(EPS)

S10 Fig Placement statistics for synthetic contigs from the chromosomal DNA associated the p4_2_1.1 plasmid. We created synthetic contigs from the chromosomal DNA (*E. coli*) by cutting at random positions. Distances between cuts were taken from a truncated exponential distribution. Attempts were made to place these synthetic chromosomal contigs on the p4_2_1.1 experimental barcode using our contig scaffolding method. The placement ratio, i.e., the number of placed contigs/total number of contigs was calculated, for different mean contig sizes in the range from 10 kbps to 80 kbps. This procedure was repeated 100 times, providing us with mean and standard deviations for the placement ratios. If chromosomal DNA and p4_2_1.1 experimental barcodes had no sequence similarity, we would expect the placement ratio to be around *p*_thresh_ = 0.01, which we seem to be below.
(EPS)

S11 Fig Contig scaffolding using synthetic contigs from a mixed sample from p4_2_1.1/ chromosomal DNA. Synthetic contigs were generated by randomly cutting the known DNA sequences for p4_2_1.1/ and the associated chromosomal DNA from *E. coli*. Compare to Fig 3 in the main text which show the same type of plots but for the pUUH plasmid. The distance between cuts were taken from a truncated exponential distribution. We then applied our contig scaffolding method (see Methods). (Top) Three typical examples of contig barcodes assembled on the consensus p4_2_1.1 barcode, here with average contig size = 24.5 kbps. (Bottom) The two ratios: the filling fraction = number of occupied pixels/total number of pixels, and the number of correctly placed contigs/total number of contigs were calculated. This was repeated for 100 random realizations of the cutting process, and mean values and associated standard deviations were calculated. We find that our method is effective at separating chromosomal and p4_2_1.1 DNA and, also, it rarely places a contig at the wrong position. The filling fraction increases with increasing contig size.
(EPS)

S12 Fig Schematic illustration of all the different ways that contigs can be placed on a circular reference barcode. Here two contigs, contigs 1 and 2, are placed on the reference barcode at all its possible positions. The reference barcode here is of length *x*_max_ = 10. Contig 1 is of length 3 pixels, and contig 2 is of length 5 pixels. Different color intensities represent different placement scores *b*_*n*,*x*_, where *n* labels contigs, and *x* denotes positions. The theoretical challenge is to maximize by sum of placement scores, satisfying the constraint that no pixel can be occupied more than once. Each contig is allowed to be placed at most one time. In seeking to maximize this sum one is also allowed *not* to place a given contig if that leads to a larger overall score.
(EPS)

S13 Fig Comparison of experimental barcodes and full theoretical plasmid barcodes. Theory barcodes were created using the transfer matrix method and consensus experimental barcode were generated by averaging individual experimental barcodes (see Sec. S.M. 2.2 in S1 Methods for details). Examples above are three of the plasmids considered in the main text: pUUH (top) and pEC005B (middle) and p4_2_1.1 (bottom). The pEC005A plasmid has 50 percent sequence similarity to pEC005B and is not shown here. Note that the general match of experiments and theory is good, but there areas in both barcodes where the experiments do not match well with the theory.
(EPS)

S14 Fig Example of Kymograph alignment using the SSDAlign algorithm. Unaligned (raw) kymograph (top), and a kymograph aligned using the SSDAalign method (bottom). The kymograph is from one of the pUUH molecules. The SSDAlign algorithm is described in Sec. S.M.2.1 in S1 Methods.
(EPS)

S15 Fig Illumina contig placement by MUMmer on the reference pUUH sequence. A search for all matches was made against the pUUH sequence. Any MUMmer alignment that covered less than 95% of the query contig was removed. Most contigs were mapped to a single location, but some small contigs, such as P13, had multiple matches which covered the entire contig, but were on positions of the reference that were not covered by other contigs. Contigs with a size greater than 10 kbps are labeled.
(EPS)

S16 Fig Placement by MUMmer on reference chromosome sequence from *Klebsiella pneumoniae*. The placement of contigs on the chromosome sequence was achieved in the same way as in Sec. S.M.5 S1 Methods. Contigs with a size greater than 80 kbps are labeled.
(EPS)

S17 Fig Sequence similarity for the best scoring complete and gap free MUMmer alignments between each reference and all contigs. The position with the highest percent of identical nucleotides in an ungapped alignment between each contig and the pUUH and chromosome reference sequences were obtained using MUMmer. The highest scoring position’s percent identity is plotted against the length of the contig. Each subplot is split in two. The upper half has a magnified scale, between 98% and 100%. The lower half ranges between 0% and 98%. (Left) Each contig is compared to the chromosome reference. (Right) Each contig is compared to the pUUH reference. There is one noticeable chromosomal contig outlier, *C*_90_, which has a roughly 2000 base pair region with high similarity to a region in the pUUH sequence, but the rest of which has low similarity. There is also a low scoring (98.2%) pUUH contig, *P*_4_. The low score is caused by a deletion in the contig relative to the pUUH sequence. The sequence similarity is between ungapped alignments, so a small proportion of the end of P4 is misaligned. When allowing for gaps, the similarity is instead 99.96%.
(EPS)

S1 File List of netropsin binding constants obtained using a procedure described in S1 Methods. (TXT)
