# Supplementary material for: Facilitated sequence assembly using densely labeled optical DNA barcodes: A combinatorial auction approach
Source: PLoS One. 2018 Mar 9;13(3):e0193900. doi: 10.1371/journal.pone.0193900 (PMC5844556; doi:10.1371/journal.pone.0193900)
Supplement: S1 Methods — Here we provide details and computational/mathematical arguments for the methods we use, together with examples. (PDF) [file pone.0193900.s001.pdf]

Supplementary Methods: Facilitated sequence assembly using densely labeled optical DNA barcodes: a combinatorial auction approach

Albertas Dvirnas [1], Christoffer Pichler[1], Callum L. Stewart[1], Saair Quaderi[1,2], Lena K. Nyberg[2], Vilhelm Müller[2], Santosh Kumar Bikkarolla[2], Erik Kristiansson[3], Linus Sandegren[4], Fredrik Westerlund[2], Tobias Ambjörnsson[1*],

1 Department of Astronomy and Theoretical Physics, Lund University, Lund, Sweden
2 Department of Biology and Biological Engineering, Chalmers University of Technology, Gothenburg, Sweden
3 Department of Mathematical Sciences, Chalmers University of Technology/University of Gothenburg, Gothenburg, Sweden
4 Department of Medical Biochemistry and Microbiology, Uppsala University, Uppsala, Sweden

* tobias.ambjornsson@thep.lu.se

Here we provide details and computational/mathematical arguments for the methods we use to produce the results in the main text, together with examples. This study has shared method/ideas of those in articles [1], [2] and [7], which we mention accordingly. All the data used for the article can be provided upon request from the authors.

S.M.1 DNA barcoding experiments and contig sequences

S.M.1.1 DNA preparation

Complete bacterial DNA (chromosomal and plasmid) was prepared by growing bacteria over night in Mueller-Hinton medium at 37 °C with shaking. DNA was extracted using the Qiagen Genomic-tip 100 according to the manufacturer's instructions. DNA was eluted in 5 ml elution buffer from the columns and precipitated by addition of 0.7 ml of isopropanol at room temperature and subsequently spooled from solution, dried and re-dissolved in 0.1x TE-buffer pH 7.0.

Plasmid DNA was separated from chromosomal DNA using the Qiagen Plasmid Midi kit according to the manufacturer's instructions. DNA was eluted from the columns with 5 ml of elution buffer and precipitated by addition of 0.7 ml of isopropanol at room temperature and subsequently centrifuged at 4500 rcf for 30 minutes followed by a wash with 70 % ethanol, dried and re-dissolved in 0.1x TE-buffer pH 7.0.

S.M.1.2 Contig sequences

Plasmid sequences and the chromosomal sequence of the *Klebsiella pneumoniae* containing pUUH239.2 have previously been determined and completely assembled using a combination of PacBio and Illumina sequencing (see [1, 8, 9]). The Illumina data was re-used here to build contigs by *de novo* assembly for mapping-experiments against barcode data. Contigs were assembled using the CLC Genomic workbench platform version 9. Default assembly parameters were used to give a standard contig set without any additional joining of contigs. Upon assembly of the contigs onto the experimental DNA barcodes [1] it was noted that the pUUH239.2 plasmid isolated for the barcode

experiments contained a spontaneous inversion between two inverted copies of IS26-elements and one contig spanning this region therefore gave a very poor match. This contig was therefore split into two single contigs at the inversion point and these were used in subsequent analyses.

S.M.1.3 Contig sequence alignment

Real contigs were obtained by Illumina sequencing of a sample containing chromosomal DNA from *Klebsiella pneumonia* and DNA from the plasmid pUUH239.2 (220 kbps long), see S.M.1.2 for details. The full contig data set contained 220 contigs, with an average length of 24.5 kbps (for a histogram of contig sizes, see S7 Fig). The sequence similarity between the contig sequences, pUUH plasmid and chromosomal DNA was investigated by aligning all Illumina contigs to the full reference sequences using MUMmer [10] (see figures S15 Fig and S16 Fig for placement results).

We found that the single lowest percent identity for an ungapped pUUH contig was 98.24 % for the contig $P4$ (fourth largest plasmid contig, see below for how we label contigs). The low score is caused by a single base pair insertion in the reference sequence compared to the contig, and if we allow a 1 base pair gap, the similarity increases to 99.96 %. Apart from this, all pUUH contigs had a sequence similarity > 99.8 % at their "true" positions, see S17 Fig.

We conclude that 203 of the contigs belong to the chromosomal DNA and 16 contigs belong to the plasmid. Based on the sequence alignment, each contig is assigned a "true" position, and the contigs are subsequently labeled as PX, CX or UX (with $X = 1, \ldots X_{\max}$ determining the order of contig lengths), so that $P1$ is the longest contig originating from the pUUH plasmid, $C1$ is the longest chromosomal contig, and UX contigs are unassigned contigs which fit neither on the plasmid nor on chromosomal DNA sequences (we have one such case in our data set). Based on the separation of the contigs into chromosomal and plasmid, we find that the average length of chromosomal contigs is 25.4 kbps, and the average of plasmid contigs is 13.5 kbps.

S.M.1.4 Optical DNA mapping experiments

In the DNA barcoding experiments, DNA was stained with YOYO-1 and netropsin, ratios 1:5 (YOYO-1) and 30:1 (netropsin) with respect to DNA, in Tris-Borate-EDTA buffer (for details see [1]). Photonicking was reduced by addition of 2% v/v Beta-mercaptoethanol to the solution before the start of the experiment. Nanochannels with dimensions of 100x150nm^2 and a length of 500 μm were used in order to stretch the DNA. Information about fabrication methods can be found in [11]. DNA was moved through microchannels from loading wells to inlets of nanochannels by using pressure-driven flow created from nitrogen gas. Plasmids were inserted into nanochannels in their circular form and subsequently linearized using light irradiation [1]. 200 frames with 100ms exposure time of each linearized plasmid molecule were obtained using an EMCCD camera (Photometrics Evolve 0.1592 um) in combination with an inverted fluorescence microscope (Zeiss AxioObserver.Z1) with 100x oil immersion objective (NA = 1.46). Additional to plasmid DNA, lambda-DNA (48502bp, New England Biolabs) was included as an internal size reference in each measurement. In total 8 lambda molecules were imaged for the pEC005A and pEC005B plasmids, 20 lambda molecules for pUUH239.2, and 6 lambda molecules for p4_2_1.1 plasmid. The size references from these measurements were 500 bp/pixel for p4_2_1.1, 592.3 bp/pixel for pUUH239.2 and 538.18 bp/pixel for the pEC005A and pEC005B plasmids.

The DNA concentration of the plasmid samples were measured using a NanoDrop. Lambda-DNA was diluted to desired concentration from a 500 ng/μl stock.

S.M.2 Generating and comparing DNA barcodes

The output from DNA barcode experiments (see Sec. S.M.1.4) is a fluorescence intensity kymograph along individual DNA molecules of four previously sequenced plasmids, pEC005A (13 kymographs), pEC005B (14 kymographs), p4_2_1.1 (35 kymographs) and pUUH (14 kymographs). In this section we show how the individual molecules are processed to make experimental barcodes (by time averaging and then computing the consensus tree), how to generate theoretical contig barcodes, and how to compare barcodes using match scores and p-values.

S.M.2.1 Aligning and time averaging of the kymographs using the SSDAlign algorithm

In the first step, kymographs are aligned using an alignment method. Previous articles used a method called WPAlign [12]. In this study we are using a sum-square based alignment method (refered to as SSDAlign), which is computationally faster (our implementation is about 4 times faster). The SSDAlign algorithm proceeds as follows:

- As a first step of the method, we generate a filtered kymograph by filtering all the rows of the kymograph with a Gaussian filter with 1.88 pixel width (this choice depends on the point spread function width and the camera lens) to convert kymograph rows into barcodes with similar correlation properties to theoretical barcodes [28].
- We use k-means clustering [29] of the filtered kymograph rows to separate the row pixels into background and molecule pixels.
- We then compute the sum square differences between the first filtered row and all the other rows, with an allowed displacement of a few pixels (equal roughly to a number of uncertain pixels at the edges of the kymograph, see S.M.2.5 for more explanation on this). For each row, we find the displacement which gives the minimal sum square difference. This gives us a number of pixels by which we have to shift each row to align it to the first row. In this way, we get an aligned kymograph, which corrects for the global positional displacement, see S14 Fig for an example of such an aligned kymograph.
- Finally, we estimate the left and right edges of the molecule by taking the average of first molecule pixels on the left and last on the right in the aligned kymograph. Using these we cut out a barcode from the time-average of the aligned kymograph, thus producing an intensity profile along a single molecule.

Note that, in contrast to WPAlign [12], the SSDAlign algorithm does not perform any local stretching operations to the kymograph.

S.M.2.2 Experimental consensus barcodes

We get a consensus barcode by averaging several individual barcodes using an adaptation of previous method [1]. As an input to this method, we have the intensity profiles which were calculated for each aligned kymograph by time-averaging. The individual barcodes are then compared against each other, the best pairs are averaged and the procedure is repeated until we get a barcode cluster (consensus tree). This tree is then “cut” based on a threshold for the best Pearson correlation coefficients. This threshold is here taken as 0 (so that we include all the barcodes in the consensus). The difference from the previous method is that we average background-mean rescaled barcodes (i.e. barcodes normalized by substracting the mean and dividing by

background-mean), which allows us to keep more information from the barcode (thus making the clustering process more accurate). The output of this method is then experimental consensus barcodes

$$\mathbf{B} = \{B_x, x = \overline{1, x_{\max}}\}$$

with lengths $x_{\max} = 128$ pixels (pEC005A, mean 127.8, standard deviation 6.7), $x_{\max} = 250$ pixels (pEC005B, mean 249.1, standard deviation 15.1), $x_{\max} = 302$ pixels (p4_2_1.1, mean 301.3, standard deviation 9.2) and $x_{\max} = 373$ pixels (pUUH, mean 372.4, standard deviation 28.1), see S13 Fig. Since we also know the length of the DNA sequences, we here use the lengths of consensus barcodes to find the real pixel/bp ratios, which are correspondingly 592, 549 and 551 bp/pixels.

S.M.2.3 Competitive binding method

We here improve the parameters used in the model introduced in [2] to produce theoretical barcodes using a DNA sequence as input. The transfer matrix method described in [2] is one of 5 possible biophysical formulations of 1-D lattice models [3]. The goal of the competitive binding method is to compute the probabilities, $p_1(i)$ and $p_2(i)$, which gives us the probability that a base-pair i is occupied by one of the monomers of the first ligand type (netropsin) or second ligand type (YOYO-1), respectively.

In the statistical physics method in [2], transfer matrices are constructed in a way so that each matrix element (m, m') corresponds to the statistical weights assigned to the combination of states where state m follows the state m' for a given base-pair i. In case of netropsin and YOYO-1 competitive binding, this simple model gives us a position dependent transfer matrix which are described as

$$T(i) = \begin{array}{r} \text{Free} \\ \text{Bound netropsin, 4th} \\ \text{Bound netropsin, 3rd} \\ \text{Bound netropsin, 2nd} \\ \text{Bound netropsin, 1st} \\ \text{Bound YOYO-1, 4th} \\ \text{Bound YOYO-1, 3rd} \\ \text{Bound YOYO-1, 2nd} \\ \text{Bound YOYO-1, 1st} \end{array} \begin{pmatrix} 1 & 0 & 0 & 0 & 1 & 0 & 0 & 0 & 1 \\ 1 & 0 & 0 & 0 & 1 & 0 & 0 & 0 & 1 \\ 0 & 1 & 0 & 0 & 0 & 0 & 0 & 0 & 0 \\ 0 & 0 & 1 & 0 & 0 & 0 & 0 & 0 & 0 \\ 0 & 0 & 0 & \mathrm{c_1 K_1(i)} & 0 & 0 & 0 & 0 & 0 \\ 1 & 0 & 0 & 0 & 1 & 0 & 0 & 0 & 1 \\ 0 & 0 & 0 & 0 & 0 & 1 & 0 & 0 & 0 \\ 0 & 0 & 0 & 0 & 0 & 0 & 1 & 0 & 0 \\ 0 & 0 & 0 & 0 & 0 & 0 & 0 & \mathrm{c_2 K_2(i)} & 0 \end{pmatrix} \quad \text{(S.M.1)}$$

Here, $\mathrm{K_1(i)}$ is the netropsin binding constant for a site beginning at base-pair i, and $\mathrm{c_1}$ is the concentration of netropsin. Similarly, $\mathrm{K_2(i)}$ and $\mathrm{c_2}$ are the binding constants and the concentration for YOYO-1.

Based on the transfer matrices above, as in [2], the total partition function Z is

$$Z = \mathbf{v}(1)^T \cdot \mathrm{T(1)} \cdot \mathrm{T(2)} \cdots \mathrm{T(N)} \cdot \mathbf{v}(\mathrm{N}+1) \quad \text{(S.M.2)}$$

where $\mathbf{v}(1)$ and $\mathbf{v}(N+1)$ are the vectors describing the boundary conditions (states at the two ends of the DNA).

Let us now derive the explicit expressions for $p_1(i)$ and $p_2(i)$ using a slightly different approach compared [2]. Following [4], if for a given base-pair i the parameter X enters the statistical weight of a given state uniquely, then the probability of this state is equal to the corresponding derivative of the partition function, multiplied by X and divided by the partition function, i.e.

$$\text{probability of states associated to X} = \frac{\mathrm{X}}{\mathrm{Z}} \frac{\partial \mathrm{Z}}{\partial \mathrm{X}} \quad \text{(S.M.3)}$$

In particular, in the transfer matrix approach, for a given base-pair i, we have a transfer matrix T(i) elements associated to all the possible parameters X, and no other matrices depend on X. There are 4 different states associated to the base-pair i bound by a ligand s (by its first, second, third or fourth monomer), corresponding to 4 off-diagonal entries in the matrix $T(i)$. Therefore the probablity of any of these is going to be the sum of the corresponding probabilities. The derivative of T(i) with respect to X can be described by a projection operator $\mathrm{O_s}$, which projects only to the states that are possible for site i. Here $\mathrm{O_1} = \mathrm{diag}(0, 1, 1, 1, 1, 0, 0, 0, 0)$ for the states associated to netropsin and $\mathrm{O_2} = \mathrm{diag}(0, 0, 0, 0, 0, 1, 1, 1, 1)$ for the states associated to YOYO-1. In this way we write

$$\sum_{i=1}^{4} X_i \frac{\partial T(i)}{\partial X_i} = O_s \cdot T(i) \tag{S.M.4}$$

Now, for a given base-pair i, the number of allowed states (configurations) for a ligand of type s is

$$\mathrm{Z_s(i)} = \sum_{\mathrm{i}=1}^{4} \mathrm{X_i} \frac{\partial \mathrm{Z}}{\partial \mathrm{X_i}} = \mathbf{v}(1)^{\mathrm{T}} \cdot \mathrm{T}(1) \cdot \mathrm{T}(2) \cdots \mathrm{T}(\mathrm{i}-1) \cdot \mathrm{O_s} \cdot \mathrm{T}(\mathrm{i}) \cdots \mathrm{T}(\mathrm{N}) \mathbf{v}(\mathrm{N}+1) \tag{S.M.5}$$

Finally, from S.M.3 and S.M.5, the probabilities that a given base-pair i has either YOYO-1 or netropsin attached to it are described as

$$p_1(i) = p_{\mathrm{netropsin}}(i) = \frac{Z_1(i)}{Z}, \quad p_2(i) = p_{\mathrm{YOYO}}(i) = \frac{Z_2(i)}{Z} \tag{S.M.6}$$

Note that p_1 and p_2 depend on position along the DNA (through binding constants $K_1(i)$ and $K_2(i)$, and concentrations c_1 and c_2). The results above are identical to the expressions given in [2], The equation for the binding probabilities S.M.6 is then written recursively for a computationally efficient competitive binding method [2], which scales with N, the total number of base-pairs.

The improvement we here make to the method above is two-fold. Our two amendments are described in detail below.

First, in [2] it was implicitly assumed that the two ligand types were in excess of the DNA. For the general case this may not be so, and one should replace the total concentrations, c_1 and c_2, in the transfer matrices above by the free concentration (concentration of non-DNA-bound ligands) [4]. To that end we first estimate the free concentrations of YOYO-1 and Netropsin, c_1^{free} and c_2^{free}, using

$$c_s^{\mathrm{free}} = c_s - c_s^{\mathrm{bound}} = c_s - \bar{\theta}_s(c_1^{\mathrm{free}}, c_2^{\mathrm{free}}) \cdot c^{\mathrm{DNA}} \tag{S.M.7}$$

Here $\bar{\theta}_s$ is the expected number of bound ligands on a single DNA molecule divided by the number of basepairs of that molecule (note that $0 \leq \bar{\theta}_s \leq [1/\lambda]$, where $\lambda = 4$ is the number of basepairs covered by a ligand for the case YOYO-1 and netropsin). Also, c^{DNA} is the concentration of DNA basepairs. Using the expressions from [4] for the expected number of bound ligands, we have

$$\bar{\theta}_s = \frac{Q_s}{Z} \tag{S.M.8}$$

where

$$Q_s = \sum_{i}^{N} \mathbf{v}(1)^T \cdot \mathrm{T}(1) \cdot \mathrm{T}(2) \cdots \mathrm{T}(\mathrm{i}-1) \cdot \mathrm{P_s} \cdot \mathrm{T}(\mathrm{i}) \cdots \mathrm{T}(\mathrm{N}) \mathbf{v}(\mathrm{N}+1) \tag{S.M.9}$$

with projector operators $\mathrm{P}_1 = \mathrm{diag}(0,0,0,0,1,0,0,0,0)$ and $\mathrm{P}_2 = \mathrm{diag}(0,0,0,0,0,0,0,0,1)$. This gives a system of two equations with two unknowns. Since these are non-linear, we solve the system numerically, by minimizing

$$\chi^2 = \min_{c_s^{\mathrm{free}}} \left(\sum_{i=1}^{2} (c_i^{\mathrm{total}} - c_i^{\mathrm{free}} - c_i^{\mathrm{bound}})^2 \right) \tag{S.M.10}$$

and make sure that the minimum occurs close to $\chi^2 = 0$. To the purpose of estimating $\bar{\theta}_{\mathrm{s}}$, we use λ-phage DNA (48502 bp) as input sequence, simply because the exact sequence content of all the DNA in the sample is not known. The concentration of DNA basepairs is experimentally estimated using a procedure described in Sec. S.M.1.4. Once the the free ligand concentrations are obtained using the steps above, these are used as the input to the transfer-matrix method. In S1 Fig we compare total and free concentrations for realistic input concentrations (typical netropsin, YOYO-1 and DNA concentrations are 6 μM, 0.04 $\mu M$and 0.2 μM, respectively).

Our second amendment to the theory barcode method from [2] is to use more accurate binding constants. To that end, we use the 5-mer intensity values of netropsin extracted from [26] to estimate all 256 possible combinations of 4-mer binding constants K_2 (see suppl.txt file). In some detail, relative fluorescence values were first extracted from [26] supplementary information plots by measuring (in pixels) the height of each percentage fluorescence bar and comparing it to the total height of the plot. All 512 5-mers reported by the paper were extracted. Only 512 5-mers should be required to know all 1024 possible combinations. Netropsin binds to double-stranded DNA, so the binding constant of one k-mer is the same as its reverse complement. Unfortunately the sequences in the paper are selected to cover all straightforward complementary permutations, not the reverse complement, so all 5-mers can not be found. Instead, all possible 4-mers are calculated as an average of the 5-mers that contain them. Note that also that in study [26], there are only single binding sites, so there is no competition (no transfer matrices are required) and there is only one ligand (netropsin). Therefore, netropsin binding constants are computed as [26]

$$K_{\mathrm{NNNN}} = \frac{P(\mathrm{NNNN})}{c^{\mathrm{free}} - P(\mathrm{NNNN}) \cdot \mathrm{c}^{\mathrm{free}}} \tag{S.M.11}$$

Here P is the binding probability determined from [26], and $NNNN$ is any of the tetramers. This provides us with new Netropsin binding constants for the competitive binding method, see S3 Fig.

We finally need an estimation of the YOYO-1 binding constant, K_2. To that end, we assume that YOYO-1 binds non-specifically to the DNA and formulate a minimization procedure for finding the optimal YOYO-1 binding constant K_2, using pEC005A and pEC005B barcodes as a training set, see S2 Fig. We find which that the optimal YOYO binding constant is $K_2 \approx 26\ (\mu M)^{-1}$. This value for the YOYO-1 binding constant and the netropsin binding constants listed the S1 File are used throughout this study.

We estimate that the improvement in correlation coefficient, when using the new binding constants (compared to the old binding constant from [2]), is around 0.06-0.07 when matching experimental consensus barcodes to the theoretical barcodes for pUUH and pEC005B.

S.M.2.4 Theoretical contig barcode generation

As an input to the theoretical contig barcode generation method, we use a DNA sequence of length k_{max} basepairs (bps) together with optical mapping related parameters provided in Sec. S.M.2.3. As an output, we get a probability vector

$$\mathbf{p} = \{p(k), k = \overline{0, k_{\mathrm{max}} - 1}\}, \tag{S.M.12}$$

which gives us the probabilities that a YOYO-1 ligand is bound to a particular basepair along the DNA. Given this probability vector, $\mathbf{p}$, a theoretical barcode is computed as a convolution of $\mathbf{p}$ with a Gaussian kernel $\boldsymbol{\phi}$ with a width of σ bps:

$$\boldsymbol{\phi} = \left\{ e^{-\frac{k^2}{2\sigma^2}}, \begin{cases} |k| \leq \lfloor \frac{k_{\max}}{2} \rfloor, k_{max} \text{ odd} \\ -\frac{k_{\max}}{2} < k \leq \frac{k_{\max}}{2}, k_{max} \text{ even} \end{cases} \right\} \tag{S.M.13}$$

here σ is 300 nm [2], but since convolution is done at the base-pair level, we use the nm/bp convertion to get the Gaussian kernel width σ in base-pairs. Conversion factors from nm to bps for previously unsequenced DNA can be obtained using the lambda-phage reference, or using the theoretical sequences, if they are known, see S.M.1. For pUUH, the conversion is 0.2690 nm/bp, and therefore $\sigma = 1115.2$ bps. For pEC005B, the conversion is 0.2954 nm/bp, and $\sigma = 1015.6$ bps.

The convolution is now written according to

$$\tilde{I}(k) = (\mathbf{p} * \boldsymbol{\phi})(k) = \sum_{i=0}^{k_{\max}-1} p(i) \cdot \phi(i-k), \; 0 \leq k \leq k_{\max} - 1 \tag{S.M.14}$$

Finally, using the camera's resolution (here, 159.2 bps/pixel), we find the conversion rates from bp to pixel, see S.M.1.4. We then interpolate to the pixel resolution to mimic the effects due to the system's optical point spread function, thus producing a pixelated theoretical contig barcode I_x, where $x = \overline{1, x_{\max}}$ labels different pixels.

S.M.2.5 Bit-weights for theoretical contig barcodes

In steps 1 and 2 in our contig scaffolding method (see the Methods section in the main text) we match a contig barcode to an experimental barcode. Due to the convolution with the PSF with width σ, certain care is required when performing such a matching. To understand why, assume we have a full DNA sequence of the form $XXXYYYYYYXXX$, where the Ys denotes a contig sequence, and the Xs denotes the remaining part of the DNA sequence. Due to the convolution nature of both experiments and theory, the X and Y regions will intermix in the experimental DNA barcode. However, using only the contig sequence, we can make no prediction of this intermixing (which occurs over distances of a few σs.). Thus, in effect, a few kbps at the ends of the theoretical contig barcode will not match the experimental consensus barcode. As in previous studies we take care of this effects by using a bit-weight function [1],

$$W_{\tilde{I}}(k) = \begin{cases} 1, 3\sigma \leq k \leq k_{\max} - 1 - 3\sigma \\ 0, \text{otherwise} \end{cases} \tag{S.M.15}$$

The size of the bit-weight window depends on the width, σ, of the point-spread function of the experimental system. We here choose the window size to be 3σ. Finally, the bit-weight function $W_{\tilde{I}}(k)$ is interpolated to pixel resolution in the same way as the theoretical barcode, thus producing a pixelated bit-weight function W_x, $x = \overline{1, x_{\max}}$.

S.M.2.6 Comparing experimental consensus and contig barcodes using a match score

As a match score between two barcodes, U and V, we use a standard statistical measure of linear dependence, the Pearson's product-moment correlation coefficient, C, defined by:

$$C(U, V) = \frac{1}{x_{\max} - 1} \sum_{x=1}^{x_{\max}} \frac{(U_x - \overline{U})(V_x - \overline{V})}{\sigma_U \sigma_V} \tag{S.M.16}$$

with $\overline{U} = \sum_x U_x/x_{\max}$, $\overline{V} = \sum_x V_x/x_{\max}$ and the corresponding standard deviations for U and V are σ_U, σ_V. In practice, the computation of the Pearson correlation coefficients is numerically evaluated by using fast Fourier transform. Since the mean values are subtracted and the standard deviations are used for normalization in Eq. (S.M.16), the Pearson correlation coefficient only measures "features" and is not sensitive to amplitude nor overall level differences between two barcodes.

Furthermore, the consensus barcode is always assumed to be intact, therefore no special care is needed for the "overlapping" bit-weight functions in the current study, and for that reason from here on we do not mention the use of bit-weights anymore, as the effect of a bit-weights on only one of the barcodes is easily understood.

S.M.2.7 Generating random barcodes

In this section we go through the steps of estimating the autocorrelation function for barcodes, and our method for computing random barcodes based on the estimated autocorrelation. The present method is similar in spirit to the approach introduced in [7].

Consider a database containing a set of M probability vectors $p_m(k)$, $m = 1, \dots, M$ at base-pair resolution. The length (in bps) of barcode m is $k_{\max,m}$ (the pixels are labeled by $k = 0, \dots, k_{\max,m} - 1$). As in [1] these M barcodes are here theory barcodes obtained using all the DNA sequences in the RefSeq plasmid database longer than 1000 base-pairs [1]. From the probability vectors we get the theoretical barcodes $\tilde{I}_m(k)$ by convolving with the point spread function (still in base-pair resolution) (see Sec. S.M.2.4). The associated Fourier transforms are

$$\hat{I}_m(\omega_{l,m}) = \hat{p}_m(\omega_{l,m}) \cdot \hat{\phi}(\omega_{l,m}) \tag{S.M.17}$$

with angular frequencies

$$\omega_{l,m} = \frac{2\pi l}{l_{\max,m}}, \quad l = 0 \dots l_{\max,m} - 1. \tag{S.M.18}$$

with $l_{\max,m} = k_{\max,m}$, i.e., there are equally many Fourier modes as there are basepairs. The autocovariance function for barcode m is defined as

$$\tilde{A}_m(k) = E \sum_{k'=0}^{k_{\max,m}-1} (\tilde{I}_m(k') - \mu_m)(\tilde{I}^*_m(k-k') - \mu^*_m) = \underbrace{E \sum_{k'=0}^{k_{\max,m}-1} \tilde{I}_m(k')\tilde{I}^*_m(k-k')}_{\tilde{r}_m(k)} - \mu^2_m \tag{S.M.19}$$

where $E(\dots)$ denotes expectation values, and μ_m is the mean value of barcode m. In Fourier-space we get

$$\hat{r}_m(\omega_{l,m}) = |\hat{\phi}(\omega_{l,m})|^2 \cdot |\hat{p}_m(\omega_{l,m})|^2 \tag{S.M.20}$$

where we used the convolution theorem. Equipped with a "database" of autocorrelation functions $\tilde{r}_m(k)$ we proceed by introducing the database-averages defined by

$$\tilde{r}(k) = \frac{1}{M} \sum_{m=1}^{M} \tilde{r}_m(k) \tag{S.M.21}$$

Ideally, we would now want to insert Eq. (S.M.20) into Eq. (S.M.21) and perform the database-average over m [7]. However, before this can be achieved, the Fourier amplitudes need be interpolated (note that the frequencies $\omega_{l,m}$ are different between barcodes, in general).

Our interpolation scheme proceed as follows: all Fourier amplitudes $|\hat{p}_m(\omega_{l,m})|$ are interpolated to the same length $L_{\max}$, and then re-normalised so that the corresponding vectors in the real space would have the same mean and standard deviation as before interpolation. To achieve this, we note that in Fourier space we have the relation for the first mode,

$$|\hat{p}_m(\omega_{0,m})| = \sum_k p_m(k) \tag{S.M.22}$$

and the Parseval's theorem,

$$\frac{1}{l_{\max,m}} \sum_l |\hat{p}_m(\omega_l)|^2 = \sum_k p_m^2(k) \tag{S.M.23}$$

We want the same relations to hold for the interpolated amplitudes $|\hat{p}_m^{\text{interp}}(\omega_l)|$ as well. Making sure that Eq. (S.M.22) is satisfied is straight-forward as we can just rescale the amplitude for the first frequency. For the second relation, we introduce $L = \frac{L_{\max}}{l_{\max,\text{m}}}$, and define a re-normalization factor

$$C_m = \sqrt{\left|\frac{L^2 \cdot \sum_l |\hat{p}_m(\omega_{l,m})|^2 - |\hat{p}_m^{\text{interp}}(\omega_0)|^2}{\sum_l |\hat{p}_m^{\text{interp}}(\omega_l)|^2 - |\hat{p}_m^{\text{interp}}(\omega_0)|^2}\right|}. \tag{S.M.24}$$

Using this factor, we then rescale

$$\hat{p}_m^{\text{interp}}(\omega_l) = \hat{p}_m^{\text{interp}}(\omega_l) \cdot C_m, \; l = 1 \ldots L_{\max} - 1 \tag{S.M.25}$$

The sequences $\hat{p}_m^{\text{interp}}(\omega_l)$ so obtained satisfy Eqs. (S.M.22) and (S.M.23).

With the interpolation scheme above in place, we can now insert Eq. (S.M.20) into Eq. (S.M.21) and perform the database-average over m in order to get a "universal" averaged database autocorrelation function. In Fourier-space the estimated database-averaged Fourier amplitudes are defined by

$$\hat{p}^{\text{est}}(\omega_l) = \sqrt{\frac{1}{M} \sum_{m=1}^{M} |\hat{p}_m^{\text{interp}}(\omega_l)|^2}. \tag{S.M.26}$$

and the autocorrelation function reads

$$\hat{r}(\omega_l) = \frac{1}{M} \sum_{m=1}^{M} |\hat{\phi}(\omega_l)|^2 |\hat{p}_m^{\text{interp}}(\omega_l)|^2 = |\phi(\omega_l)|^2 |\hat{p}^{\text{est}}(\omega_l)|^2 \tag{S.M.27}$$

for an arbitrary choice of the angular frequencies $\omega_l = \frac{2\pi l}{L_{\max}}, l = 0 \ldots L_{\max} - 1$.

We are now in a position to compute random barcodes. As in [7], we use phase randomization to that purpose. First, we interpolate the database-averaged Fourier amplitudes to a new length $k_{\max}$, so that it corresponds to the length of a barcode in base-pairs.

As a next step, we draw $\lfloor k_{\max}/2 \rfloor$ uniformly distributed random numbers, α, and symmetrically multiply the amplitude of $\hat{p}^{\text{est}}(\omega_l)$ by a set of random phases $(1 \; e^{i\alpha_1} \ldots e^{i\alpha_{\lfloor k_{\max}/2 \rfloor}} \; e^{i\alpha_{\lfloor k_{\max}/2 \rfloor - 1}} \ldots e^{i\alpha_1})$, here $e^{i\alpha_{\lfloor k_{\max}/2 \rfloor - 1}} = 1$ if $\lfloor k_{\max}/2 \rfloor = k_{\max}/2$. In this way we get a Fourier-transformed random binding probability vector

$$\hat{p}^{\text{rand}}(\omega_l) = \hat{p}^{\text{est}}(\omega_l) e^{i\alpha_l} \tag{S.M.28}$$

A corresponding random barcode is generated by the operation

$$\hat{I}^{\text{random}}(\omega_l) = \hat{p}^{\text{rand}}(\omega_l) \cdot \hat{\phi}(\omega_l), \quad 0 \le l \le k_{\max} - 1 \tag{S.M.29}$$

which is then transformed into the real space by taking the inverse Fourier transform. Finally, by pixelating the basepair resolution barcode, we get a random barcode I_x^{random}, used below.

Note that the procedure above guarantees that the covariance estimate, see Eq. (S.M.27), for random barcodes remains the same as for the original database-average covariance (since $|\hat{I}(\omega_l)|^2 = |I^{\text{random}}(\omega_l)|^2$). In this sense, the present method generates "realistic looking" random barcodes.

S.M.2.8 Distributions of match scores for random barcodes

For this study, we have chosen the Pearson correlation coefficients (see Sec. S.M.2.6) as a match scores between two barcodes. For two normally distributed random data sets $\mathbf{U} = (U_1, \ldots, U_\nu)$ and $\mathbf{V} = (V_1, \ldots, V_\nu)$ with independent elements, the probability density function of such match scores has been derived in [13] and [14]. For DNA barcodes, however, due to, for instance, the blurring effect from the point spread function, intensity values at different pixels are not independent quantities. Following the same line of thought as in [2] we deal with this effect by simply replacing the parameters in the distribution for the Pearson correlation coefficient (ν and λ, see below) by effective ones (ν_{eff} and λ_{eff}) which are determined by fitting. The justification for this is that for short range correlations we can divide pixels into independent entities [2].

With the above approach for dealing with correlation in mind, we denote the Beta function by $B(w,z)$, i.e. $B(w,z) = \int_0^1 x^{w-1}(1-x)^{z-1}dx$. Then the probability density function (PDF) for C in this case is (see [13] and [14])

$$f_\nu(C) = \frac{(1-C^2)^{\frac{\nu-4}{2}}}{B(\frac{1}{2}, \frac{\nu}{2}-1)}, -1 \leq C \leq 1 \tag{S.M.30}$$

which belongs to a class of the first kind of generalized beta distributions [15].

In our case, we slide contig barcodes across an experimental barcode and seek the *largest* correlation coefficient. Hence, rather than the PDF for C we are interested in the PDF for the largest C in a set. To that end, suppose now that we have computed a series of λ correlation coefficients, $\{C_1, \ldots, C_\lambda\}$. Then, the cumulative distribution function for the maximum of these,

$$\hat{C} = \max\{C_1, \ldots, C_\lambda\} \tag{S.M.31}$$

is given by [16]

$$\rho_{\nu,\lambda}(\hat{C}) = \left(\int_{-1}^{\hat{C}} f_\nu(x)dx\right)^\lambda \tag{S.M.32}$$

Here the integral $\int_{-1}^{\hat{C}} p_\nu(x)dx$ can be computed by making a substitution $x^2 = t,\ 2xdx = dt$. This then leads to the following expression for the cumulative distribution function, which depends on two parameters, ν and λ:

$$\rho_{\nu,\lambda}(\hat{C}) = \left(\frac{1}{2}\left(1 + (-1)^{sgn(C)} I_{C^2}(\frac{1}{2}, \frac{\nu}{2} - 1)\right)\right)^\lambda \tag{S.M.33}$$

Here $sgn(q) = \begin{cases} 1, q \geq 0 \\ 0, q < 0 \end{cases}$, and $I_x(a,b)$ is the regularised incomplete beta function,

$$I_x(a,b) = \frac{\int_0^x t^{a-1}(1-t)^{b-1}dt}{B(a,b)} \tag{S.M.34}$$

The probability density function for $\hat{C}$ can be computed from the cumulative distribution function by taking the derivative, i.e.

$$\phi_{\nu,\lambda}(\hat{C}) = \frac{d\rho_{\nu,\lambda}(\hat{C})}{d\hat{C}} \tag{S.M.35}$$

Further we consider only the cases where $\hat{C} > 0$. Then

$$\phi_{\nu,\lambda}(\hat{C}) = \lambda \left(\frac{1}{2}\left(1 + I_{C^2}(\frac{1}{2}, \frac{\nu}{2} - 1)\right)\right)^{\lambda-1} \frac{(1-\hat{C}^2)^{\frac{\nu-4}{2}}}{B(\frac{1}{2}, \frac{\nu}{2} - 1)} \tag{S.M.36}$$

In previous studies [2] and [7], it was assumed that for large ν, the PDF for the match scores is well approximated by a Gaussian function (as guaranteed by the central limit theorem). However, for smaller ν this is no longer the case. Therefore in this paper the PDF for $\hat{C}$ as given in Eq. (S.M.36) is used instead.

S.M.2.9 Maximum likelihood estimation in distribution fit

Our procedure for estimating the effective parameters ν_{eff} and λ_{eff} (see Sec. S.M.2.8) is described in this section. For compactness of notation, we set $\nu = \nu_{\text{eff}}$ and $\lambda = \lambda_{\text{eff}}$ below.

In brief, first R random barcodes (labeled by $r = 1, \ldots R$) of a predetermined length are generated, using the method described in Sec. S.M.2.7. We here use $R = 1000$. Each of the R random barcodes is then compared to the experimental barcode, using the Pearson correlation coefficient, for every possible position on the experimental barcode, and the maximum coefficient, $\hat{C}_r$, is stored. These maximum correlation coefficients are used to create a histogram. This histogram is fitted to the functional form provided in Sec. S.M.2.8, see Eq. (S.M.36), in order to determine ν and λ.

Our fitting procedure uses the maximum likelihood method, which is described in detail below for our particular form of fitting function. The probability density function for a random variable $\hat{C}$, conditioned on parameters ν and λ, is denoted by $\phi(\hat{C}|\nu,\lambda) := \phi_{\nu,\lambda}(\hat{C})$. The joint density function, also called the likelihood function, $L(\nu, \lambda|\ \hat{C})$ of R independent identically distributed observations is

$$\phi(\hat{C}_1, \ldots, \hat{C}_R|\ \nu, \lambda) = \prod_{r=1}^{R} \phi(\hat{C}_r|\ \nu, \lambda) = L(\nu, \lambda|\ \hat{C}) \tag{S.M.37}$$

To determine the parameters of the extreme value distribution, we work with

$$\log(L(\nu, \lambda|\ \hat{C})) = \sum_{r=1}^{R} \log \phi_{\nu,\lambda}(\hat{C}_r|\ \nu, \lambda) = \tag{S.M.38}$$

$$R\log(\lambda) + (\lambda - 1)\sum_{r=1}^{R} \log(\left(\frac{1}{2}\left(1 + I_{\hat{C}_r^2}(\frac{1}{2}, \frac{\nu}{2} - 1)\right)\right)) + \tag{S.M.39}$$

$$\frac{\nu-4}{2}\sum_{r=1}^{R} \log((1 - \hat{C}_r^2) - R\log(B(\frac{1}{2}, \frac{\nu}{2} - 1)) \tag{S.M.40}$$

where we used Eq. (S.M.36). The necessary conditions for maximising $\log(L(\nu, \lambda|\ \hat{C}))$ are

$$\begin{cases} \frac{\partial L(\nu,\lambda|\ \hat{C})}{\partial \lambda} = 0, \\ \frac{\partial L(\nu,\lambda|\ \hat{C})}{\partial \nu} = 0. \end{cases} \tag{S.M.41}$$

We have

$$\frac{\partial L(\nu,\lambda|\,\hat{C})}{\partial \lambda} = \frac{R}{\lambda} + \sum_{i=1}^{R} \log\left(\frac{1}{2}\left(1 + I_{\hat{C}_r^2}(\frac{1}{2}, \frac{\nu}{2} - 1)\right)\right) = 0. \tag{S.M.42}$$

From this equation we get

$$\lambda = \frac{1}{\log(2) - \frac{1}{R}\sum_{r=1}^{R} \log\left(1 + I_{\hat{C}_r^2}(\frac{1}{2}, \frac{\nu}{2} - 1)\right)} \tag{S.M.43}$$

Similarly, we can derive the equation for ν, to get

$$\frac{\partial L(\nu,\lambda|\,\hat{C})}{\partial \nu} = \texttt{term1} + \texttt{term2} + \texttt{term3} = 0 \tag{S.M.44}$$

where

$$\texttt{term1} = (\lambda - 1)\sum_{r=1}^{R} \frac{d}{d\nu} \log(1 + I_{\hat{C}_r^2}(\frac{1}{2}, \frac{\nu}{2} - 1)) \tag{S.M.45}$$

$$\texttt{term2} = \frac{1}{2}\sum_{r=1}^{R} \log((1 - \hat{C}_r^2) \tag{S.M.46}$$

$$\texttt{term3} = \frac{R}{2}(\psi(\frac{\nu-1}{2}) - \psi(\frac{\nu-2}{2})) \tag{S.M.47}$$

where ψ is the digamma function, i.e. $\psi(x) = \frac{\Gamma'(x)}{\Gamma(x)}$. The solution to Eqs. (S.M.43) and (S.M.44) gives us estimates for $\nu = \nu_{\text{eff}}$ and $\lambda = \lambda_{\text{eff}}$.

S.M.2.10 Hypothesis test and p-value for barcode matching

We now define a hypothesis test to quantify the significance of the matches of contig barcodes on an experimental barcode (as compared to matches to random barcodes). To that end, for a given contig barcode n of a certain size we use the procedure in the previous subsection to calculate the distribution fit parameters, ν_{eff} and λ_{eff}, by matching random contig barcodes of the same size to the experimental barcode, including subsequent fitting. We also calculate observed match scores, $C_{n,x}$, by comparing the actual contig barcode to the experimental barcode at all positions (including flips). A p-value is then defined as:

$$\text{p-value} = \int_{C_{n,x}}^{1} \phi_{\nu,\lambda}(\hat{C}')d\hat{C}' = 1 - \int_{-1}^{C_{n,x}} \phi_{\nu,\lambda}(\hat{C}')d\hat{C}' = 1 - \rho_{\nu,\lambda}(C_{n,x}) \tag{S.M.48}$$

where $\phi_{\nu,\lambda}(\hat{C})$ is the fitted PDF, and $\rho_{\nu,\lambda}(\hat{C})$ is given explicitly in Eq. (S.M.33). Then, if the p-value is less than the significance level of p_{thresh} (here we use $p_{\text{thresh}} = 0.01$) we say that the contig barcode is significantly different from a random barcode. Therefore, for that given position, the contig barcode is deemed to fit well along the experimental barcode.

S.M.3 Contig scaffolding using a combinatorial auction algorithm

In this section we introduce a combinatorial auction method, which is then used to place a set of contig barcodes along an experimental barcode without overlap. We begin with giving some definitions and examples of the problem. Then we discuss some of the

related algorithms, which were developed previously to solve similar combinatorial problems. Finally we explain in detail the combinatorial auction method used in this paper. In the contig scaffolding method, we assume that there are no inserts or deletions in the experimental consensus barcode. This will allow us to consider a contig as "continuous", and we will either find a placement for the whole contig, or we will not place it at all.

S.M.3.1 Brute-force approach

Before getting into the combinatorial auctions algorithm, it is useful to explain why we use a combinatorial auction algorithm to solve the problem, rather than use a "brute-force" approach where all possible contig positions are tested.

Consider all contigs which passed the length threshold (see step 3 of the contig scaffolding algorithm in the main text). For each of these contigs we compute placement scores $b_{n,x}$. These contigs could then, in principle, be placed using a brute force algorithm. Each of the contigs labeled by $n = 1, \dots N$ can be placed on the experimental barcode in $x_{\max}$ different ways (not taking orientation into account), where $x_{\max}$ is the length of the experimental barcode. This gives us $x_{\max}^N$ different possibilities for placement of contigs, see Supplementary Figure S11. In the brute force approach, we then look through these to find the cases with no overlap. Then the placement with the maximum placement score (defined below) gives us the solution to the contig scaffolding problem. While this gives the correct solution to the contig scaffolding problem, the computational time scales exponentially with N, thus rendering it very impractical for larger N values (and $x_{\max}$). In the subsequent subsections, we therefore introduce a dynamic programming type approach to the present maximization problem.

S.M.3.2 Formulation of the contig placement challenge as a combinatorial auction problem

We will approach the problem of contig scaffolding by formulating it as a special case, the interval bidding problem [17], of a combinatorial auction problem (CAP).

Assume that we have a reference barcode, which is $x_{\max}$ pixels long. The reference barcode is circular, therefore the pixels can be placed on a circle, see Supplementaary Figure S11. We also have N contigs and contig n is d_n long. A given contig n has a placement score (bid value) $b_{n,x}$ associated to it, when placed with its last pixel at position x on the reference barcode ($x = 1, \dots, x_{\max}$). A bit value $b_{n,x} = 0$ corresponds to a "no bid" ($p_{n,x} < p_{\text{thresh}}$, see main text). The challenge at hand is to combine the placement scores $b_{n,x}$ from different contigs in such a way that the sum of their values, $\sum b_{n,x}$, is maximized. To make the problem mathematically precise, let $y_{n,x} = 1$ if contig n is included in the final combination at the location x, and $y_{n,x} = 0$ if it is not. Then the *winner determination problem* is the following optimization problem

$$\max \sum_{n=1}^{N} \sum_{x=1}^{x_{\max}} b_{n,x} y_{n,x} \tag{S.M.49}$$

with the following set of constraints:

$$\begin{cases} \forall\ x' = \overline{1, x_{\max}}\ , \quad \sum_{n=1}^{N} \left(\sum_{\substack{x=\overline{1,x_{\max}} \\ x' \in \text{x'th location}}} y_{n,x} \right) \leq 1 \\ \forall\ n = \overline{1, N}\ \ \sum_{x=1}^{x_{\max}} y_{n,x} \leq 1 \end{cases} \tag{S.M.50}$$

The first constraint means that each pixel can be assigned only to one contig, i.e. we can have no overlaps. The second constraint means that each contig can be placed at

most once. The solution to this problem is a matrix $\mathbf{Y}$, which stores all the information needed about the placed contigs and the placement positions.

A simplified version of this problem was considered and solved in [18] and [19]. In this simplified version, each contig would be allowed to have only one placement score (bid only once), and further it could be assumed that the placement is on a line, rather than a circle. A solution algorithm to this problem, running in $O(x_{\max}^2)$ time and using dynamical programming approach, was suggested in aforementioned articles. In [19] an algorithm for solving the problem without simplification of bidding on a line was introduced. Such an algorithm is solved in the same way as the first one after dividing the problem into $x_{\max}$ subproblems. Therefore the original algorithm has to be run $x_{\max}$ times, and hence it takes $O(x_{\max}^3)$ time. In Van Hoesel's paper [18], in addition to the simplified version of the problem, a dynamic programming solution for a general problem, running in $O(N2^N x_{\max}^2)$ time, was also suggested. The idea of the algorithm behind this solution is the same as the one we will use when defining a contig scaffolding algorithm. Our contribution here is that we are able to decrease the factor 2^N above by using additional structure of the contig assignment problem.

S.M.3.3 An improved exact combinatorial auction algorithm for the interval bidding problem

As we mentioned before, the idea behind our algorithm is based on the dynamical programming type approach to the interval bidding problem described in [18]. Assume that the experimental barcode we place the contigs on is linear (any circular problem can be reduced to $x_{\max}$ linear subproblems, as explained below). Let U be the subset of contigs, i.e. $U \subset U_{\text{all}} = \{n_1, \ldots, n_N\}$, and define by $\mathrm{F_{overall}(U, x-1)}$ the value of the optimal placement score of first $x-1$ pixels, i.e., $\mathrm{F_{overall}(U, x-1)}$ is the optimal placement score for a contig placement problem involving only pixels $\overline{1, x-1}$ with $0 \leq x \leq x_{\max}$. Then the optimal placement value for x pixels, $\mathrm{F_{overall}(U, x)}$ can be found by

$$F_{\text{overall}}(U, x) = \max \begin{cases} F_{\text{overall}}\,(U, x-1) \\ \max_{n \in U}\left(F_{\text{overall}}\,(U \setminus \{n\}, x - d_n + 1) + b_{n,x}\right) \end{cases} \tag{S.M.51}$$

with initial conditions $F_{\text{overall}}(U, 0) = 0$ and $F_{\text{overall}}(\emptyset, x) = 0$. The method in [18] uses Eq. (S.M.51) recursively as a way to generate the optimal score for the final problem, i.e., to calculate $F_{\text{overall}}(U_{\text{all}}, x_{\max})$, where U_{all} is the full set of contigs (bidders). We improve on this method in two ways. Firstly, it can often happen that there are no new placement scores when we move from placing x pixels to placing $x+1$ pixels (see Eq. (S.M.55)). For such cases our algorithm therefore directly jump to the pixel where new placement scores are introduced. Secondly, while the number of possible subsets of contigs $U \subset \{n_1, \ldots, n_N\}$ is 2^N, the number of subsets at a given step in the algorithm is in general smaller (see Sec. S.M.3.4 for an example of this, as well as S.M.3.5 for further explanation).

We now briefly explain all the steps of the algorithm used to find the optimal placement value of all the contigs, which we call $F_{\text{overall}}(U_{\text{all}}, x_{\max})$. As an input to this algorithm we have contigs $n_1, \ldots, n_N$, length of the reference barcode $x_{\max}$, length of contigs d_n, and associated placement scores $b_{n,x}$. The steps of the algorithm are then the following:

1. *Creating a matrix of placement scores.* We represent all the placement scores $b_{n,x}$ in the form of a matrix B,

$$\mathrm{B} := \mathrm{b_{n,x}}|_{\mathrm{n=1\ldots N, x=1\ldots x_{max}}} = \begin{pmatrix} b_{1,1} & b_{1,2} & \ldots & b_{1,x_{\max}} \\ \vdots & \ddots & \ddots & \vdots \\ b_{N,1} & b_{N,2} & \ldots & b_{N,x_{\max}} \end{pmatrix} \tag{S.M.52}$$

Here each row $n = \overline{1,N}$ contains all the placement scores for the contig n to be placed along the reference barcode. The column x represents placement on the reference barcode, when the last pixel of the contig is placed at x.

2. *Mapping to m linear subproblems.* We reduce the problem with a circular reference barcode to $x_{\max}$ simpler subproblems. This is done by "cutting" the reference barcode at each of $x_{\max}$ possible pixels, in this way creating a new matrix of placement scores, $\mathrm{B_{x'}}$,

$$\mathrm{B_{x'}} := \begin{pmatrix} b_{1,x} & \ldots & b_{1,x_{\max}} & \ldots & b_{1,x-1} \\ \vdots & \ddots & \ddots & \ddots & \vdots \\ b_{N,x} & \ldots & b_{N,x_{\max}} & \ldots & b_{N,x-1} \end{pmatrix} \tag{S.M.53}$$

Since the problem is now linear, not all of the placements are possible, and we have $b_{n,x} = 0$, if $x < d_n$. For example, if the first contig has $d_1 = 5$, then $b_{1,1} = b_{1,2} = b_{1,3} = b_{1,4} = 0$.

3. *Dynamical algorithm.* Starting from the first pixel x, which has $b_{n,x} \neq 0$ for some $n \in \overline{1,N}$, we compute the $F_{\mathrm{overall,x'}}(U_x, x)$. This is done using Eq. (S.M.51). Here the number of U_x, subsets of contigs, depends on the number of pixels x. Here U_x differs from U used before, since before we considered all the subsets of U_{all}, but now U_x is smaller. When only one pixel is present, $x = 1$, we have that U_x will contain only one-contig subsets for contigs n for which $b_{n,x} \neq 0$. For $x > 1$, there will be more possible subsets, as subsets of two or more contigs will be possible too. The optimal placement value of the algorithm will then be

$$F_{\mathrm{overall}}(U_{\mathrm{all}}, x_{\max}) = \max_{x'} F_{\mathrm{overall,x'}}(U_{x_{\max}}, x_{\max}) \tag{S.M.54}$$

4. *Backtracking.* We start from $U_{x_{\max}}$, the subset of contigs for which the optimal placement value $F_{\mathrm{overall}}(U_{\mathrm{all}}, x_{\max})$ is reached. These are the contigs that are placed by the combinatorial auction algorithm. Then we just trace backwards through $F_{\mathrm{overall,x'}}(U_{x_{\max}}, x_{\max})$, using Eq. (S.M.51), to find out at which pixel each contig has been placed. This gives us the optimal placement for each contig as an outcome, i.e. we find the matrix $\mathbf{Y} = \{y_{n,x}, n = \overline{1,N}, x = \overline{1,x_{\max}}\}$.

S.M.3.4 Example

We here provide a simple example of the working of our combinatorial contig placement algorithm, introduced in Sec. S.M.3.3. Consider 5 contigs ($N = 5$) to be placed on a linear reference barcode with 6 pixels ($x_{\max} = 6$). We consider only the linear subproblem with start pixel at $x' = 1$ (the remaining subproblems are dealt with in an identical way). Contig sizes are $d_n|_{n=\overline{1,5}} = 1, 2, 3, 4, 5$, and we are given placement scores $b_{1,3} = 2$, $b_{1,4} = 5$, $b_{2,3} = 3$, $b_{2,5} = 3$, $b_{3,1} = 2$,$b_{3,3} = 4$, $b_{4,1} = 2$, $b_{4,4} = 4$,$b_{4,6} = 3$, $b_{5,1} = 1$, $b_{5,2} = 8$, $b_{5,3} = 3$, $b_{5,4} = 4$ and $b_{5,6} = 1$. By applying a brute force approach, i.e. manually going through all allowed contig configurations, it is straightforward (although tedious) to show that the optimal contig placement is to place contig 1 at position $x = 4$ and that contig 3 covers the pixels $x = \overline{1,3}$. The associated optimal placement score is 9. We now show that this result can be recovered using our method from Sec. S.M.3.3.

1. Firstly, we represent the placement scores by a matrix of placement score,

$$B = \begin{pmatrix} 0 & 0 & 2 & 5 & 0 & 0 \\ 0 & 0 & 3 & 0 & 3 & 0 \\ 2 & 0 & 4 & 0 & 0 & 0 \\ 2 & 0 & 0 & 4 & 0 & 3 \\ 1 & 8 & 3 & 4 & 0 & 1 \end{pmatrix}$$

2. In the first linear subproblem (and here there is only one subproblem, since reference barcode is linear), the matrix of placement scores becomes

$$B_1 = \begin{pmatrix} 0 & 0 & 2 & 5 & 0 & 0 \\ 0 & 0 & 3 & 0 & 3 & 0 \\ 0 & 0 & 4 & 0 & 0 & 0 \\ 0 & 0 & 0 & 4 & 0 & 3 \\ 0 & 0 & 0 & 0 & 0 & 1 \end{pmatrix} \tag{S.M.55}$$

Note that the first two columns of B_1 are all zero. This happens because contigs $3, 4$ and 5 are longer than 2 pixels, and neither of them can be placed just on the first two pixels, while contigs 1 and 2 had no placement scores for these pixels from the formulation of the problem. Therefore, when computing the optimal placement score, we are able to start immediately from $x = 3$.

3. The first x for which there is n such that $b_{n,x} \neq 0$ is $x = 3$,

$$B_1 = \begin{pmatrix} 0 & 0 & 2 & 5 & 0 & 0 \\ 0 & 0 & 3 & 0 & 3 & 0 \\ 0 & 0 & 4 & 0 & 0 & 0 \\ 0 & 0 & 0 & 4 & 0 & 3 \\ 0 & 0 & 0 & 0 & 0 & 1 \end{pmatrix}$$

In the third column of B_1 (olive color), we have first, second, and third contigs with non-zero placement values $b_{1,3}, b_{2,3}, b_{3,3}$. Therefore there will be three subsets of the contigs in U_3. Using Eq. (S.M.51), we find the values of $F_{\text{overall},1}(U, 3)$, $F_{\text{overall},1}(U, 3)$ =(2, 3, 4), where $U \in U_3 =$ (1), (2), (3) . Here by red color we label new subsets of contigs and corresponding maximum placement scores.

The next pixel to consider is $x = 4$. Then

$$B_1 = \begin{pmatrix} 0 & 0 & 2 & 5 & 0 & 0 \\ 0 & 0 & 3 & 0 & 3 & 0 \\ 0 & 0 & 4 & 0 & 0 & 0 \\ 0 & 0 & 0 & 4 & 0 & 3 \\ 0 & 0 & 0 & 0 & 0 & 1 \end{pmatrix}$$

and the contigs to consider are $n = 1$ and $n = 4$. This gives us $U \in U_4 =$ (1), (2), (3), (4), (1,2), (1,3) and $F_{\text{overall},1}(U, 4)$ =(5 ,3, 4, 4 , 8 , 9). Here by orange color we label the placement score value that has changed from the value for previous pixel.

Next pixel to consider is $x = 5$,

$$B_1 = \begin{pmatrix} 0 & 0 & 2 & 5 & 0 & 0 \\ 0 & 0 & 3 & 0 & 3 & 0 \\ 0 & 0 & 4 & 0 & 0 & 0 \\ 0 & 0 & 0 & 4 & 0 & 3 \\ 0 & 0 & 0 & 0 & 0 & 1 \end{pmatrix}$$

Only $n = 2$ contig has non-zero placement value here, and we compute $\mathrm{U} \in \mathrm{U}_5 = (1), (2), (3), (4), (1,2), (1,3), (2,3)$ and $F_{\text{overall}}(U, 5) = (5, 3, 4, 4, 8, 9, 7)$

Finally, for $x = 6$, we have

$$\mathrm{B}_1 = \begin{pmatrix} 0 & 0 & 2 & 5 & 0 & 0 \\ 0 & 0 & 3 & 0 & 3 & 0 \\ 0 & 0 & 4 & 0 & 0 & 0 \\ 0 & 0 & 0 & 4 & 0 & 3 \\ 0 & 0 & 0 & 0 & 0 & 1 \end{pmatrix}$$

and $\mathrm{U} \in \mathrm{U}_6 = (1), (2), (3), (4), (5), (1,2), (1,3), (2,3)$, and $F_{\text{overall}}(U, 6) = (5, 3, 4, 4, 1, 8, 9, 7)$

4. Finally, we see that $F_{\text{overall}}(\mathbf{Y}) = 9$, since that is the maximum of $F_{\text{overall}}(U, 6)$. To find $\mathbf{Y}$, we do simple backtracking through step 3 of the algorithm. The optimal value 9 was assigned for $n = 4$, and corresponds to the subset $\{1, 3\}$. Contig 3 had no placement score for $n = 4$ pixel ($b_{3,4} = 0$), so contig 1 was placed on this pixel with placement score $b_{1,4} = 5$. Tracing back, we get that the first contig is placed on $x = 4$, and the third contig is placed on $x = \overline{1,3}$, and the optimal placement value is 9. Therefore $y_{1,4} = 1$, and it remains to find placement of contig 3, with the placement score $9 - 5 = 4$. Moving back by one pixel, when $n = 3$, contig 3 has placement score $b_{3,3} = 4$. Therefore, it is placed at pixels $x = \overline{2,3}$, and $y_{3,3} = 1$. This gives the combinatorial contig auction algorithm's outcome, matrix $\mathbf{Y}$, for the linear problem we were considering, i.e.

$$\mathbf{Y} = \begin{pmatrix} 0 & 0 & 0 & 1 & 0 & 0 \\ 0 & 0 & 0 & 0 & 0 & 0 \\ 0 & 0 & 1 & 0 & 0 & 0 \\ 0 & 0 & 0 & 0 & 0 & 0 \\ 0 & 0 & 0 & 0 & 0 & 0 \end{pmatrix}$$

S.M.3.5 Validity and speed of the new algorithm

Here we discuss the computational times associated with our algorithm for solving the *winner determination problem* for interval bidding from the previous subsections. Explanations are based on a source-nodes-sink graph, which we can draw for the *winner determination problem* considered here [20]. Then our algorithm is just a dynamical solution to the shortest path problem on this graph. From the graph formulation of the problem one sees that the maximum number of nodes for each pixel is 2^N, where N is the number of contigs. Therefore in the worst case the algorithm performs as well as the previous algorithm. However, in the cases of interest we often do much better than the worst case. The first improvement that we make is that we do not need to go through all the pixels (as seen in Eq. (S.M.55)). Secondly, we do not consider all the subsets of contigs.

For example, if the experimental barcode is $x_{\max}$ pixels long, but only M of the contigs can be placed at the same time, then rather than a scaling $2^{x_{\max}}$, the computational time scales as $\sum_{k=1}^{M} \binom{x_{\max}}{k}$. For example, if we have $N = 100$ contigs, and the reference barcode is $x_{\max}$ pixels long, but the smallest contig is of $\lfloor x_{\max}/10 \rfloor$ pixels length, then the number of nodes to go through is at worst $\sum_{k=1}^{10} \binom{100}{k} \approx 1.7 \cdot 10^{13}$, a dramatic improvement from $2^{100} \approx 1.3 \cdot 10^{30}$. In the example given in the previous section, the number of different contig subsets to consider was only 8. If we have five contigs as we had in the example the worst case would give us $2^5 = 32$ subsets. This also illustrates the argument about contig lengths. Since the sum of the lengths of three shortest contigs is 6 (1+2+3), there can be no subsets of more than

three bidders, and there could be only one subset with three bidders (but that did not even happen in the example in the previous subsection). This greatly reduced the space of elements to consider. Furthermore, we use sparse matrix interpretation to put all the possible placement scores into one matrix, and the row index has one to one correspondence to the subsets of contigs.

In practice, for the type of reference barcode length ($x_{\max} \approx 373$ pixels, pUUH) of interest here, we tested the computational times by randomly cutting the chromosomal DNA and pUUH into pieces of 24.5 kbps length on average and then attempting to place those contigs (with $p_{\text{thresh}} = 0.01$) on the pUUH contig barcode. We find that we can solve this task in on average less than one second on a modern desktop computer (a single Intel(R) Core(TM) i7-2600K CPU @ 3.40GHz processor) using our Matlab implementation.

We here mention that there has been a lot of further development of generalizations, inexact approaches to the solution of this problem, see [21–25]. The use of these techniques would allow us to consider the cases also for larger N. For example, if we used Lagrangian relaxation, our problem could be reduced to the optimization problem of the simplified version of the problem. While these techniques are not exact and might not converge in some cases (or converge to some local minima), this gives us an outlook of other possibilities of solving the contig scaffolding problem, if the exact approach introduced in the next subsection would run into computational speed limitations.

References

1. Nyberg LK, Quaderi S, Emilsson G, Karami N, Lagerstedt E, Müller V, et al. Rapid identification of intact bacterial resistance plasmids via optical mapping of single DNA molecules. Scientific Reports. 2016;6.

2. Nilsson AN, Emilsson G, Nyberg LK, Noble C, Stadler LS, Fritzsche J, et al. Competitive binding-based optical DNA mapping for fast identification of bacteria-multi-ligand transfer matrix theory and experimental applications on Escherichia coli. Nucleic acids research. 2014; p. gku556.

3. Teif VB, Rippe K. Calculating transcription factor binding maps for chromatin. Briefings in bioinformatics, 2011, 13.2: 187-201.

4. Teif VB. General transfer matrix formalism to calculate DNA-protein-drug binding in gene regulation: application to O R operator of phage λ. Nucleic acids research, 2007, 35.11: e80.

5. Satz AL., Bruice, TC. Synthesis of fluorescent microgonotropens (FMGTs) and their interactions with dsDNA. Bioorganic and medicinal chemistry 2000; 8(8): 1871-1880.

6. Larsson A et al. Characterization of the binding of the fluorescent dyes YO and YOYO to DNA by polarized-light spectroscopy. Journal of the American Chemical Society 1994; 116(19): 8459-8465.

7. Müller V, Karami N, Nyberg LK, Pichler C, Torche Pedreschi PC, Quaderi S, et al. Rapid Tracing of Resistance Plasmids in a Nosocomial Outbreak Using Optical DNA Mapping. ACS Infectious Diseases 2016;2(5): 322-328.

8. Brolund A, Franzén O, Melefors Ö, Tegmark-Wisell K, Sandegren L. Plasmidome-analysis of ESBL-producing Escherichia coli using conventional typing and high-throughput sequencing. PLoS One. 2013;8(6):e65793.

9. Sandegren L, Linkevicius M, Lytsy B, Melhus Å, Andersson DI. Transfer of an Escherichia coli ST131 multiresistance cassette has created a Klebsiella pneumoniae-specific plasmid associated with a major nosocomial outbreak. Journal of Antimicrobial Chemotherapy. 2011; p. dkr405.

10. Kurtz S, Phillippy A, Delcher AL, Smoot M, Shumway M, Antonescu C, et al. Versatile and open software for comparing large genomes. Genome biology. 2004;5(2):R12.

11. Persson F, Tegenfeldt JO. DNA in nanochannels—directly visualizing genomic information. Chemical Society Reviews. 2010;39(3):985–999.

12. Noble C, Nilsson AN, Freitag C, Beech JP, Tegenfeldt JO, Ambjörnsson T. A fast and scalable kymograph alignment algorithm for nanochannel-based optical DNA mappings. PloS one. 2015;10(4):e0121905.

13. Anderson-Cook CM. An introduction to multivariate statistical analysis. Journal of the American Statistical Association. 2004;99(467):907–909.

14. Johnson NL, Kotz S, Balakrishnan N. Continuous univariate distributions, vol. 2 of wiley series in probability and mathematical statistics: applied probability and statistics; 1995.

15. McDonald JB, Xu YJ. A generalization of the beta distribution with applications. Journal of Econometrics. 1995;66(1):133–152.

16. David HA, Nagaraja HN. Order Statistics. 2003;.

17. De Vries S, Vohra RV. Combinatorial auctions: A survey. INFORMS Journal on computing. 2003;15(3):284–309.

18. Van Hoesel S, Müller R. Optimization in electronic markets: examples in combinatorial auctions. Netnomics. 2001;3(1):23–33.

19. Rothkopf MH, Pekeč A, Harstad RM. Computationally manageable combinational auctions. Management science. 1998;44(8):1131–1147.

20. Sandholm T, Suri S. Improved algorithms for optimal winner determination in combinatorial auctions and generalizations. In: AAAI/IAAI; 2000. p. 90–97.

21. Nguyen T, Peivandi A, Vohra R. Assignment problems with complementarities. Journal of Economic Theory. 2016;165:209–241.

22. ul Hassan U, Curry E. Efficient task assignment for spatial crowdsourcing: A combinatorial fractional optimization approach with semi-bandit learning. Expert Systems with Applications. 2016;58:36–56.

23. Michalak T, Rahwan T, Elkind E, Wooldridge M, Jennings NR. A hybrid exact algorithm for complete set partitioning. Artificial Intelligence. 2016;230:14–50.

24. Zhou Y. Improved multi-unit auction clearing algorithms with interval (multiple-choice) knapsack problems. In: International Symposium on Algorithms and Computation. Springer; 2006. p. 494–506.

25. Fisher ML. The Lagrangian relaxation method for solving integer programming problems. Management science. 2004;50(12_supplement):1861–1871.

26. Boger DL et al. A simple, high-resolution method for establishing DNA binding affinity and sequence selectivity. Journal of the American Chemical Society 2001;123(25): 5878–5891.

27. Goossens DR, Spieksma FCR. Exact algorithms for the matrix bid auction. Computers operations research 2009 36(4): 1090–1109.

28. Torche, PC, et al. Noise reduction in single time frame optical DNA maps. Plos One 2017 12(6): e0179041.

29. Hartigan JA, Wong MA. Algorithm AS 136: A k-means clustering algorithm. Journal of the Royal Statistical Society. Series C (Applied Statistics) 1979 28(1): 100–108.
