# Supplementary material for: Facilitated sequence assembly using densely labeled optical DNA barcodes: A combinatorial auction approach
Source: PLoS One. 2018 Mar 9;13(3):e0193900. doi: 10.1371/journal.pone.0193900 (PMC5844556; doi:10.1371/journal.pone.0193900)
Supplement: S1 Table — (PDF) [file pone.0193900.s002.pdf]

Table S.1. Comparison between match scores (maximum Pearson correlation coefficients, $\hat{C}$) using the new competitive binding parameters and the old method from Ref. [30] in the main text.

Experimental barcode	pEC005A	pEC005b	pUUH	p4_2_1.1
Length (kbp)	70	138	221	152
$\hat{C}$, new theory	0.9647	0.9322	0.9417	0.9138
$\hat{C}$, old theory	0.9526	0.8623	0.9115	0.8751

S1 Table.
